# Long non‐coding RNAs are significantly associated with prognosis and response to therapies in gastric cancer

**DOI:** 10.1002/ctm2.421

**Published:** 2021-06-06

**Authors:** Min‐Kyue Shin, Jungmin Kim, Dachan Kim, Sung Hwan Lee, Ji‐Hyun Shin, Yun Seong Jeong, Bo Hwa Sohn, Jimin Kim, Seong‐Ryong Kim, Jaffer A. Ajani, Ju‐Seog Lee, Jae‐Ho Cheong

**Affiliations:** ^1^ Yonsei University College of Medicine Seoul Korea; ^2^ Brain Korea 21 PLUS Project for Medical Science Yonsei University College of Medicine Seoul Korea; ^3^ Department of Systems Biology and Department of GI medical Oncology The University of Texas MD Anderson Cancer Center Houston Texas USA; ^4^ Catholic University College of Medicine Seoul Korea; ^5^ Department of Surgery Yonsei University Health System Seoul Korea; ^6^ Yonsei University College of Medicine Seoul Korea; ^7^ Department of Biochemistry and Molecular Biology Seoul Korea; ^8^ Department of Biomedical Systems Informatics Yonsei University College of Medicine Seoul Korea

**Keywords:** chemotherapy, gastric cancer, immunotherapy, long non‐coding RNA (lncRNA), prognosis, ZNF667‐AS1

**ABBREVIATIONS**
EP: epithelial phenotype
GC: gastric cancer
GSEA: gene set enrichment analysis
lncRNAs: long non‐coding RNAs
MP: mesenchymal phenotype
MSI: microsatellite instability
TCGA: The Cancer Genome Atlas

Dear Editor,

We address the heterogeneity of clinical outcome in gastric cancer (GC) by applying a systems‐level characterization of the long noncoding RNA (lncRNA). We identified molecular subtypes predictive of response to standard treatments and demonstrated therapeutic potential of targeting lncRNA for the refractory subtype.

Hierarchical clustering analysis of lncRNA expression in the The Cancer Genome Atlas (TCGA) stomach adenocarcinoma cohort resulted in six distinct clusters, which were named the LNC6 subtypes (Figure S1A). Owing to the small sample size and short follow‐up period in the TCGA cohort, prognostic associations of the LNC6 subtypes were evaluated in independent public cohorts (*n *= 1,933; Table S1). Because lncRNA expression data from RNA sequencing are available for only TCGA among large‐scale cohorts, we used LNC6 subtype‐specific mRNA gene signature (Figures 1A and 1B). Bayesian compound covariate predictor algorithm was adopted to construct a prediction model,^1^ and receiver operating characteristic (ROC) analysis of the predicted probability confirmed its robustness resulting in area under the ROC curves (AUCs) greater than 0.9 for all six subtypes (Figure S2). The L6A and L6F subtypes were associated with poor prognoses, followed by the L6B and L6D subtypes, while the L6C and L6E subtypes were associated with good prognoses (Figure 1C; see also Table S2 for pairwise comparison results). LNC6 subtypes also demonstrated differences in clinicopathological and molecular characteristics (Table S3; Figure S3) – notably, more than two‐thirds of L6F tumors were Lauren diffuse type, while about 5%–10% of L6B and L6C tumors were diffuse type. In addition, most of L6A and L6D patients were from Western countries, which might account for the shrinkage of the two subtypes in the test cohorts.

**FIGURE 1:**
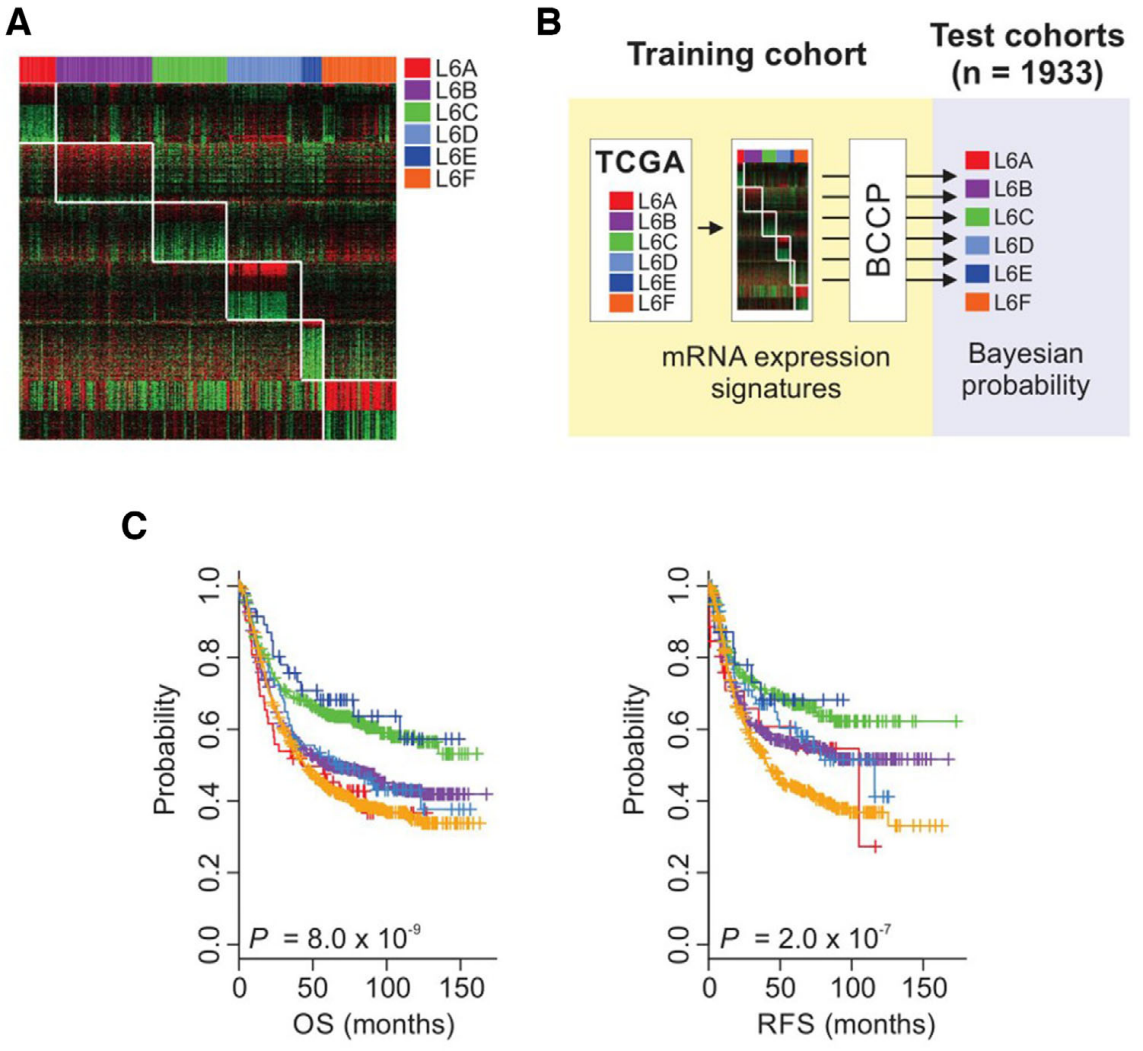
Prognostic association of LNC6 subtypes. (A) mRNA expression signature specific to LNC6 subtypes in the TCGA cohort (*n* = 258). Subtype‐specific mRNA expression signatures were identified using multiple two‐class *t*‐tests (*p* < 0.001), yielding few hundred genes for each subtype. The top 200 mRNAs were selected for each subtype according to the log ratio. (B) Schematic diagram for prediction model. Samples in the test cohorts were assigned to one of the six subtypes according to the Bayesian probability scores. (C) Kaplan–Meier plots with global *p*‐value from log‐rank test of overall survival and recurrence‐free survival of patients in the test cohorts (*n* = 1,933). In pairwise comparisons, the L6C demonstrated significantly shorter overall survival than all the other groups except the L6E, and the L6F showed significantly shorter recurrence‐free survival than the L6B and L6C (adjusted *p*‐value can be found in Table S2

We examined the association of LNC6 subtypes with chemo‐response in a merged cohort where about half of the patients had received standard adjuvant chemotherapy (Figures 2A and 2B).^2^, ^3^ Among the major three subtypes comprising 90% of this cohort, L6B patients exhibited significant benefit from chemotherapy, while L6C and L6F patients did not. We also examined the association of LNC6 subtypes with response to pembrolizumab by directly analyzing lncRNA expression from the raw RNA sequencing data of a phase 2 clinical trial (*n* = 45).^4^ The prediction model was constructed based on the expression data of subtype‐specific lncRNAs in the TCGA cohort (Figure S1B and Table S4). L6C probability was a positive predictor of clinical response to immune checkpoint blockade, whereas L6F probability was a negative predictor (Figures S4A and S4B). Although the predicted probability of the L6E subtype could not stratify the responders and non‐responders, gene set enrichment analysis (GSEA) of lncRNAs specifically upregulated in the L6E subtype successfully stratified them (Figure S4C). Furthermore, we explored the relationship of LNC6 subtypes with other molecular subtypes reported in previous studies (Figures 2C‐2F).^2^, ^3^, ^5^, ^6^ The poor prognostic L6F subtype largely overlapped with the genomically stable, epithelial‐mesenchymal transition (EMT), mesenchymal phenotype, and invasive subtypes, all of which have been associated with poor clinical outcome. Enrichment of L6C with the microsatellite instability (MSI) subtype is well matched with its negative predictive value for chemotherapy.^7^ In line with this, several L6F and L6C‐specifc lncRNAs have previously been associated with poor clinical outcome and chemoresistance (Supplementary Discussion). Meanwhile, L6B was enriched with the chromosomal instability and proliferative subtypes, supporting association between chemosensitivity and proliferative signature. L6E subtype was 100% Epstein–Barr virus (EBV) subtype, and this is also in good agreement with favorable prognoses and response to pembrolizumab in MSI‐high and EBV‐positive tumors. Taken together, consideration of the lncRNA expression pattern may add predictive value for response to adjuvant chemotherapy and immune checkpoint blockade.

**FIGURE 2:**
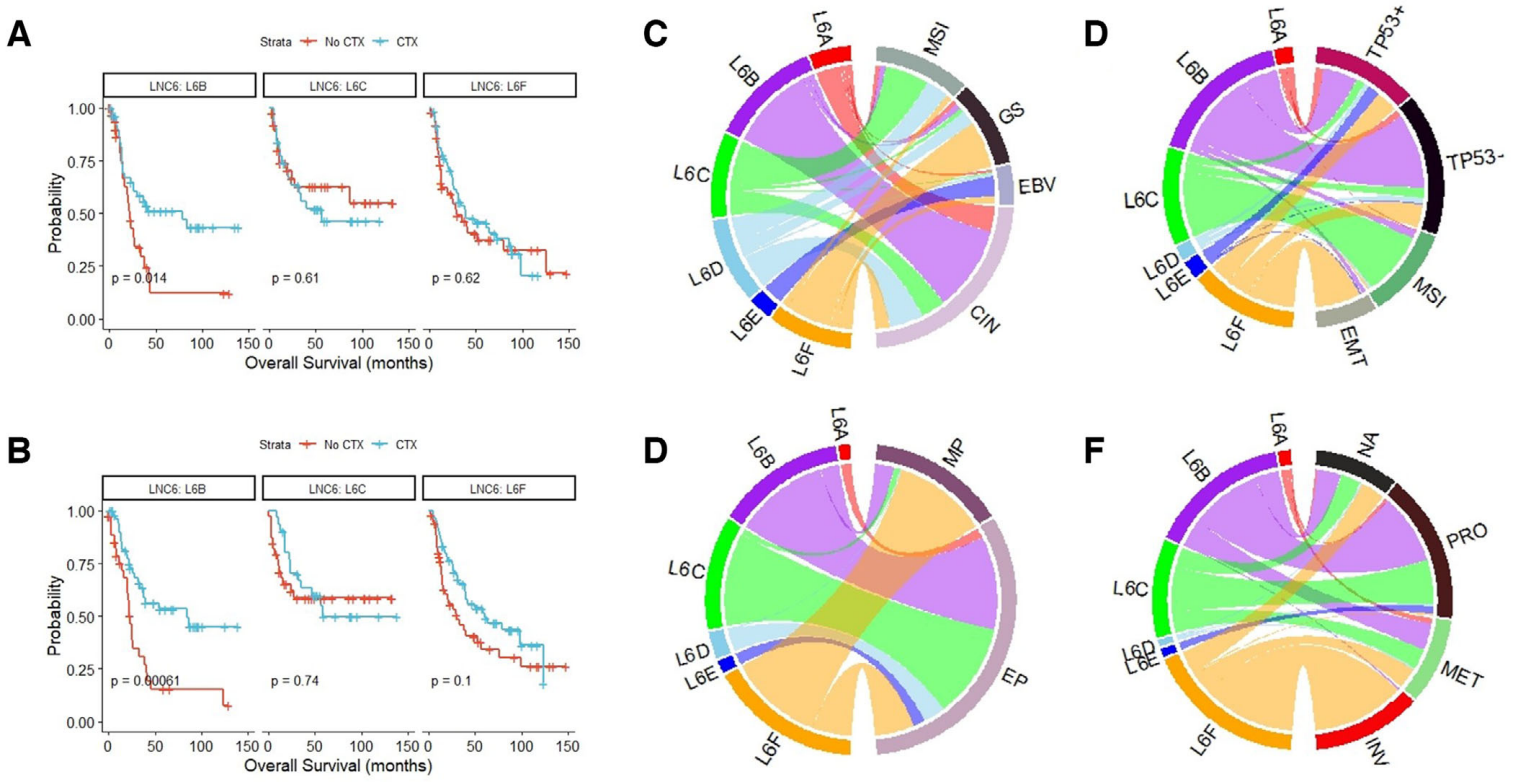
Association of LNC6 subtypes with response to chemotherapy and other molecular subtypes. (A and B) Kaplan–Meier plots of overall survival and recurrence‐free survival among patients who received adjuvant chemotherapy (CTX) and those who did not, for L6B (*n* = 90), L6C (*n* = 70), and L6F (*n* = 112) subtypes. Patients pooled from GSE13861, GSE15459, and GSE26942 with AJCC stage II, III, or IV disease without distant metastasis were included in the analysis. (C‐F) Chord diagram for the LNC6 subtypes and other classifications corresponding to each cohort. Each chord represents a sample overlapping between the two subtypes. (C) TCGA cohort. (D) GSE62254. (E) GSE13861 and GSE26942. (F) GSE15459. Abbreviations: CIN, chromosomal instability; EBV, Epstein–Barr virus; EMT, epithelial‐mesenchymal transition; EP, epithelial phenotype; GS, genomically stable; INV, invasive; MET, metabolic; MSI, microsatellite instability; MP, mesenchymal phenotype; PRO, proliferative

Moreover, we carried out bioinformatics analyses and took advantage of transcriptomic data from other studies to establish the biological implications of the LNC6 subtypes. GSEA and ingenuity pathway analysis revealed biological pathways and regulators characterizing each subtype (Figure S5; Table S5; Supplementary Discussion). In agreement with the activation of pathways associated with stemness, transcription factors overexpressed during the early stomach development were upregulated in the L6F subtype (Figure S6).^8^ Conversely, S‐phase‐enriched lncRNAs were downregulated in the L6F subtype, supporting correlation between quiescence and stem‐like characteristics (Figure S7).^9^ Such expression pattern of biological signatures might account for the bad clinical outcome consistently seen in the L6F subtype.

We investigated therapeutic approaches for the L6F subtype based on gene silencing experiment and pharmacogenomic analysis. First, we examined expression level of five L6F subtype‐specific lncRNAs in six GC cell lines using RT‐qPCR (primers listed in Table S6). Based on EMT phenotype characterization in a previous study,^10^ the average expression level was higher in the EMT subtype cell lines for all five lncRNAs, among which ZNF667‐AS1 achieved significance despite small sample number (Table S7). Then, we tested the functional association between ZNF667‐AS1 and mesenchymal/stem‐like characteristics with knockdown and overexpression studies (Figures 3A and 3B). Knockdown of ZNF667‐AS1 (ENSG00000166770.6) by siRNA in the EMT subtype cell lines decreased their migratory and invasive activity, as well as sphere formation under anchorage‐independent growth (Figure 3C). In agreement with this, silencing ZNF667‐AS1 increased the expression of an epithelial marker (E‐cadherin) and decreased the expression of mesenchymal markers (N‐cadherin and vimentin) (Figure 4A). Moreover, this increased sensitivity to drugs commonly used for GC treatment – oxaliplatin and 5‐FU (Figure 4B). On the other hand, overexpression of ZNF667‐AS1 in the non‐EMT subtype cell lines induced mesenchymal marker expression and chemoresistance, while reducing their epithelial marker expression (Figures 4A and 4B). In addition, we looked into two independent pharmacogenomic datasets of GC cell lines to identify drugs that could specifically target the L6F subtype. By performing correlation analysis between drug sensitivity AUC values and L6F probability from lncRNA expression, we found three drugs that had negative correlation with significance in both datasets ‐ YM155, PI‐103, and Obatoclax (Figure 4C). Previous studies reported the selective efficacy of these drugs against the mesenchymal and stem‐like GC (Supplementary Discussion). Our study has some limitations ‐ the retrospective nature of the clinical data and the lack of animal studies.

**FIGURE 3:**
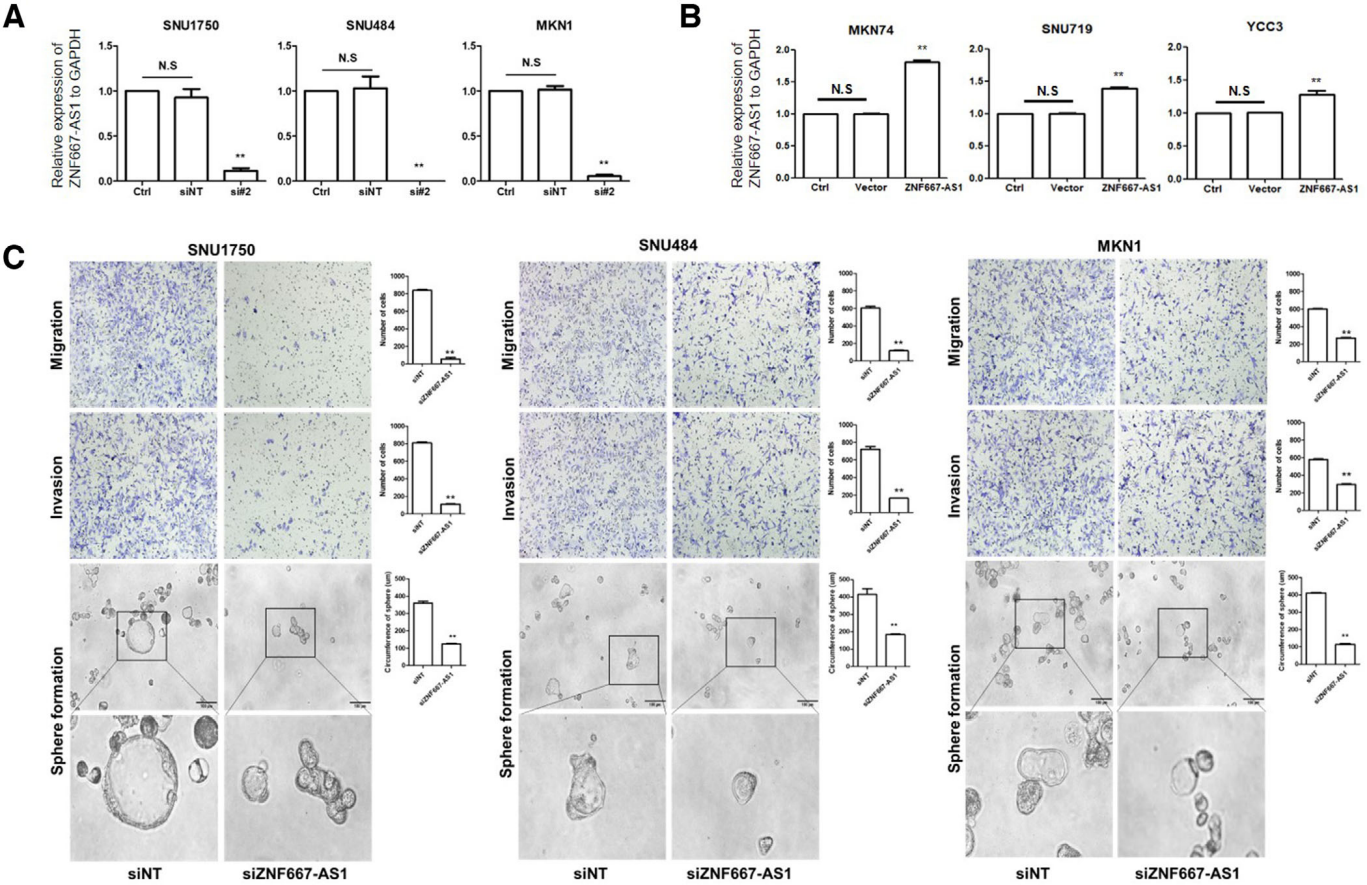
Mesenchymal/stem‐like phenotypes mediated by ZNF667‐AS1, a L6F subtype‐specific lncRNA. (A and B) Examination of ZNF667‐AS1 via real‐time PCR after transfection of (A) the EMT subtype GC cell lines with siRNA (siNT for non‐target; si#2 for ZNF667‐AS1) and (b) the non‐EMT subtype GC cell lines with vector (vector only as control; overexpression vector for ZNF667‐AS1). (C) Representative images and results of migration, invasion, and sphere formation assays from siRNA experiment on EMT‐subtype GC cell lines. Statistical bar graphs show the average results from three independent experiments. (*t*‐test, **p < 0.05; *n* = 3). The microscope images are magnified by *200 and *600

**FIGURE 4:**
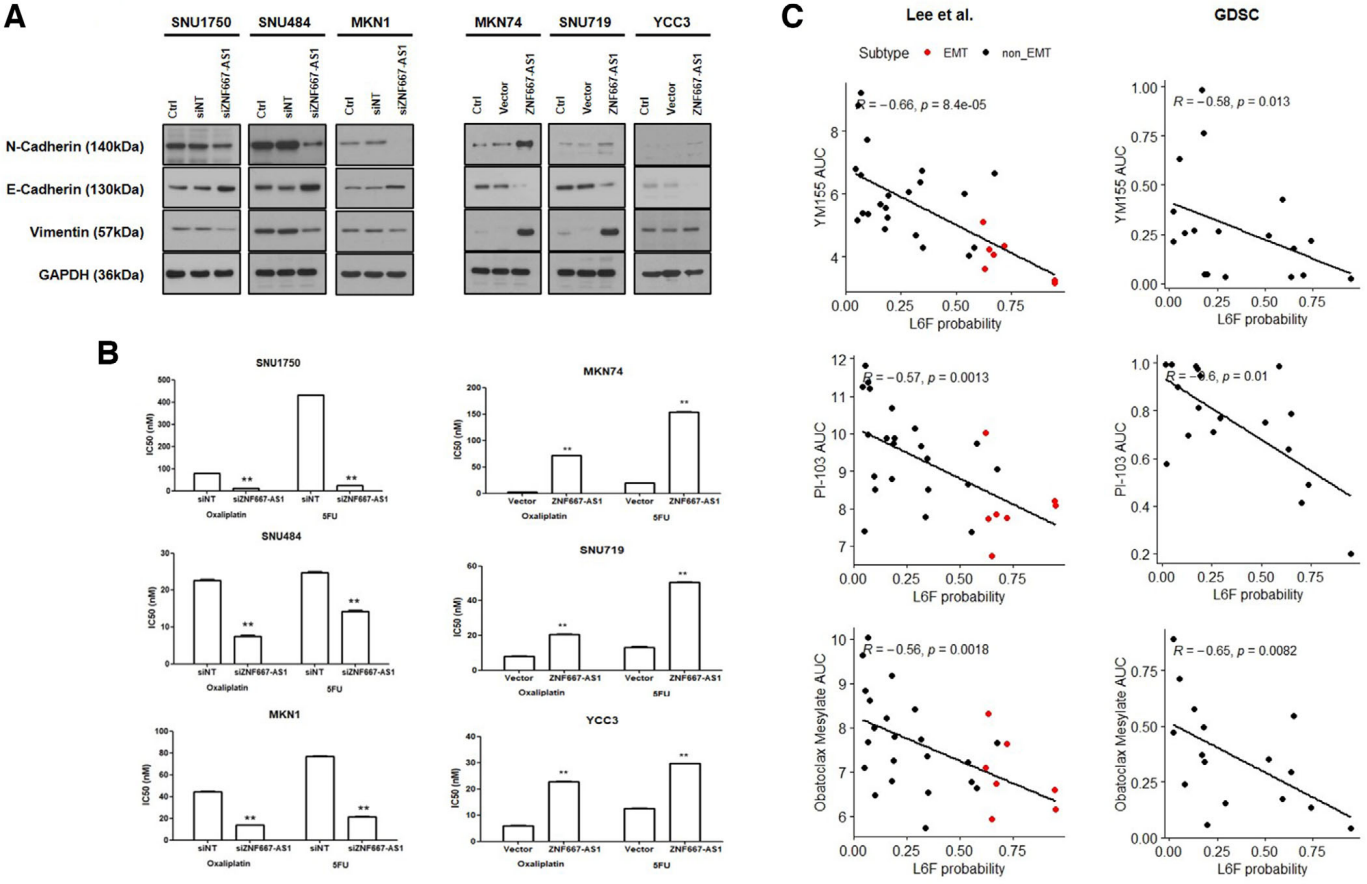
EMT marker expression and drug sensitivities associated with ZNF667‐AS1. Drug repurposing approach for the L6F subtype. (A) Western blot analyses of the indicated EMT marker proteins in siRNA‐transfected EMT subtype GC cell lines and vector‐transfected non‐EMT subtype GC cell lines. (B) Half maximal inhibitory concentration (IC50) from the MTS assay of oxaliplatin and 5‐FU in the siRNA‐transfected EMT subtype GC cell lines or vector‐transfected non‐EMT subtype GC cell lines. (C) Spearman correlation analysis between area under the dose‐response curve (AUC) values and the L6F probability predicted from lncRNA expression in two independent pharmacogenomic datasets of GC cell lines. Seven cell lines (Hs746T, SNU1750, MKN1, SK4, SNU484, SNU668, and YCC11) with previously confirmed mesenchymal phenotypes are classified as the EMT subtype in the Lee et al dataset. Abbreviations: GDSC, The Genomics of Drug Sensitivity in Cancer Project

In conclusion, we demonstrated clinical implication of the lncRNA‐based stratification and biological association of a specific lncRNA in GC. Significant association of lncRNA expression with prognosis and therapeutic responses indicate that it could be used to elaborate precision medicine for GC. Moreover, functional association of a lncRNA with clinically relevant phenotypes supports the notion of lncRNA‐targeting therapeutics.

## CONFLICT OF INTEREST

The authors declare that they have no competing interests.

## DATA AVAILABILITY STATEMENT

All microarray or RNA‐seq data used in this study are acquired from previous studies as described in text.

## Supporting information

Supporting information
