# Supplementary material for: Long non‐coding RNAs are significantly associated with prognosis and response to therapies in gastric cancer
Source: Clin Transl Med. 2021 Jun 6;11(6):e421. doi: 10.1002/ctm2.421 (PMC8181196; doi:10.1002/ctm2.421)
Supplement: Supplementary file 1 — Supporting information [file CTM2-11-e421-s001.docx]

**
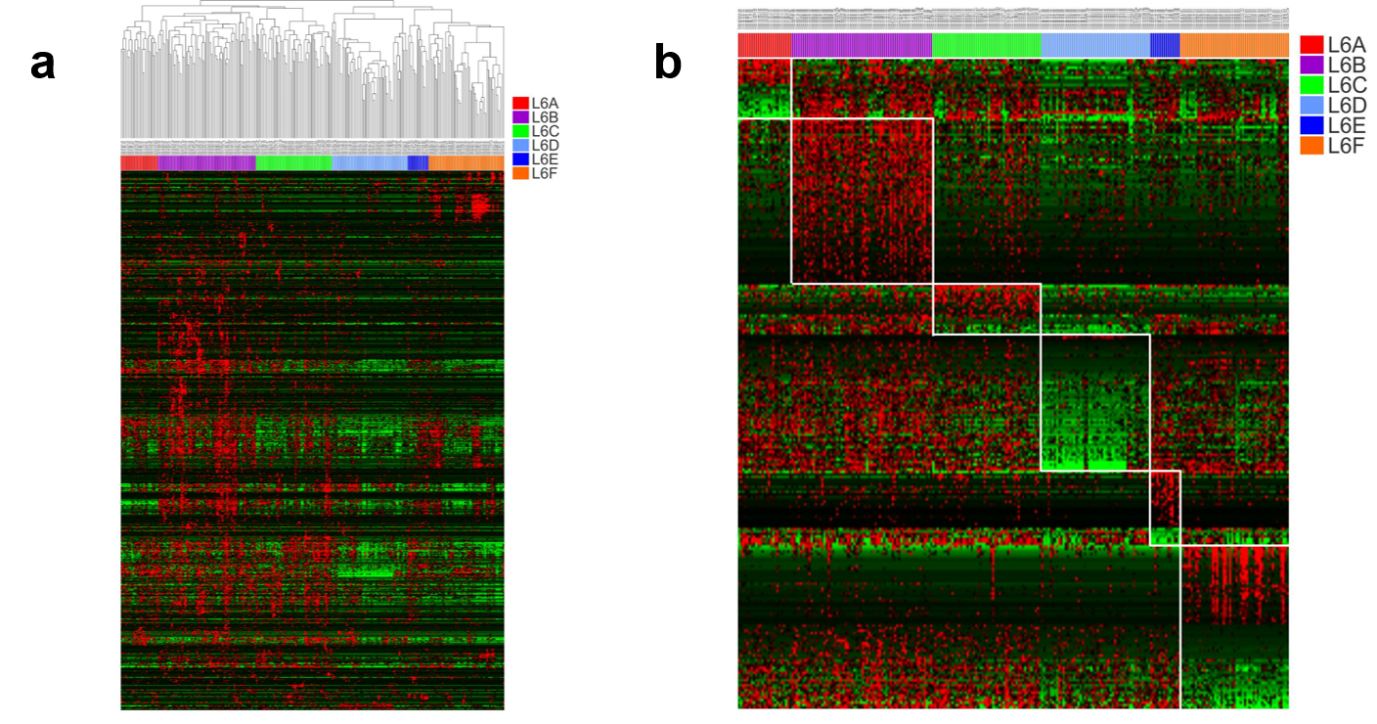
**

**Supplementary Figure S1. Hierarchical clustering and differential expression analysis of lncRNA data from STAD tissues in the TCGA cohort (n = 258).**

(a) Genes with at least 0.5-fold difference in expression levels (log2 scale) relative to the median value across tissues in at least seven tissues were selected for hierarchical clustering analysis (1,001 lncRNAs). (b) LncRNA expression signature specific to the LNC6 subtypes in the TCGA cohort. Multiple two-class t-tests were performed for all possible combinations of the six subtypes to identify subtype-specific lncRNAs. Only lncRNAs with significant differences (P < 0.05) in expression in all five possible comparisons were considered subtype-specific. The data are presented in matrix format: rows represent individual genes and columns represent each tissue. Each cell in the matrix represents the expression level of a gene feature in an individual tissue. Red and green cells reflect relatively high and low expression levels, respectively, as indicated in the scale bar (log2 transformed scale).

**
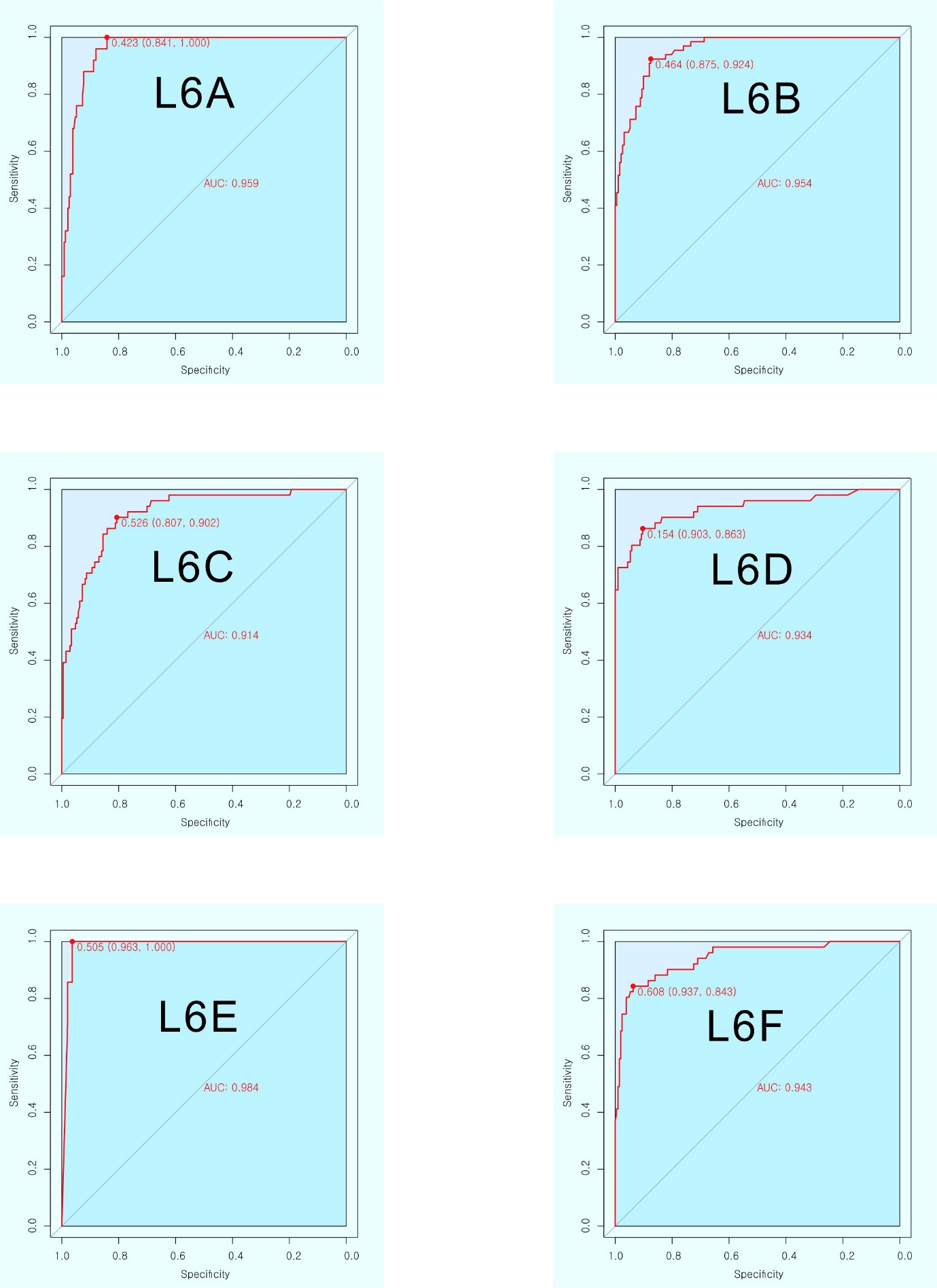
**

**Supplementary Figure S2. ROC analysis of predicted probabilities for each LNC6 subtype in TCGA cohort.**

**
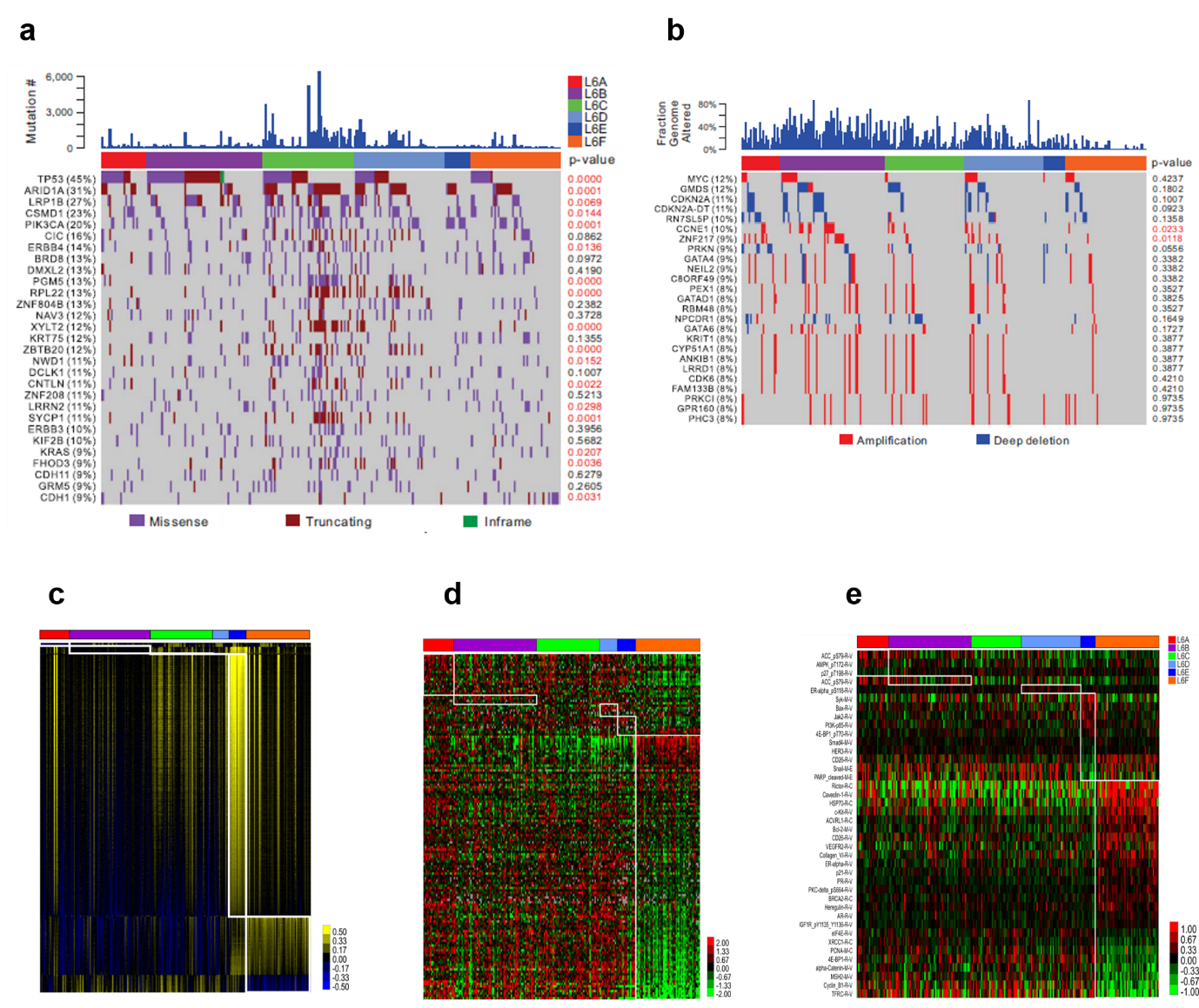
**

**Supplementary Figure S3. Multi-omics characterization of LNC6 subtypes in TCGA cohort.**

(a) Significantly mutated genes, identified by MutSigCV, are filtered by the q value and ranked by the frequency (left). Mutation colour indicates the class of mutation. Chi-square test was performed to identify subtype specific gene mutations. (b) Bars represent fraction of copy number altered genome. Significantly copy number altered genes are filtered by the q value and ranked by the frequency (left). Chi-square test was performed to identify subtype specific copy number alterations. (c-e) Multiple 2-sample t-tests were carried out to identify subtype-specific methylation of DNA and expression of miRNA and protein in the TCGA data set. (c) DNA methylation of which standard deviation less than 0.15 were ignored. (d) miRNAs of which missing value greater than 20% of cohort were ignored.

**
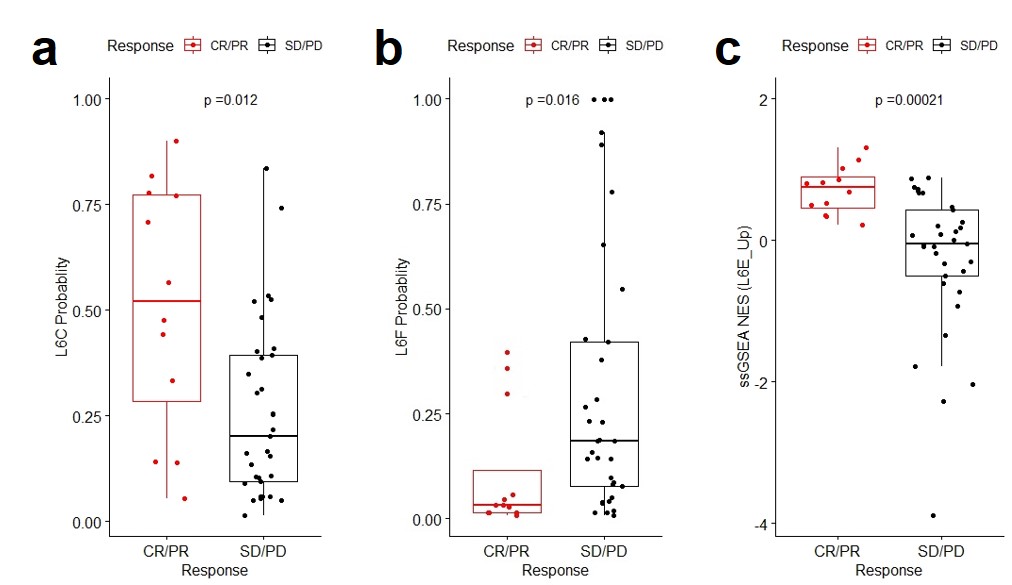
**

**Supplementary Figure S4. Immunotherapy response and the LNC6 subtypes (*n* = 45).**

(a-b) Predicted probability of the L6C and L6F subtypes among responders and non-responders. (c) Normalized enrichment score from the single-sample gene set enrichment analysis (ssGSEA) of lncRNAs upregulated in the L6E subtype among responders and non-responders. CR, complete response; PR, partial response; SD, stable disease; PD, progressive disease.

**
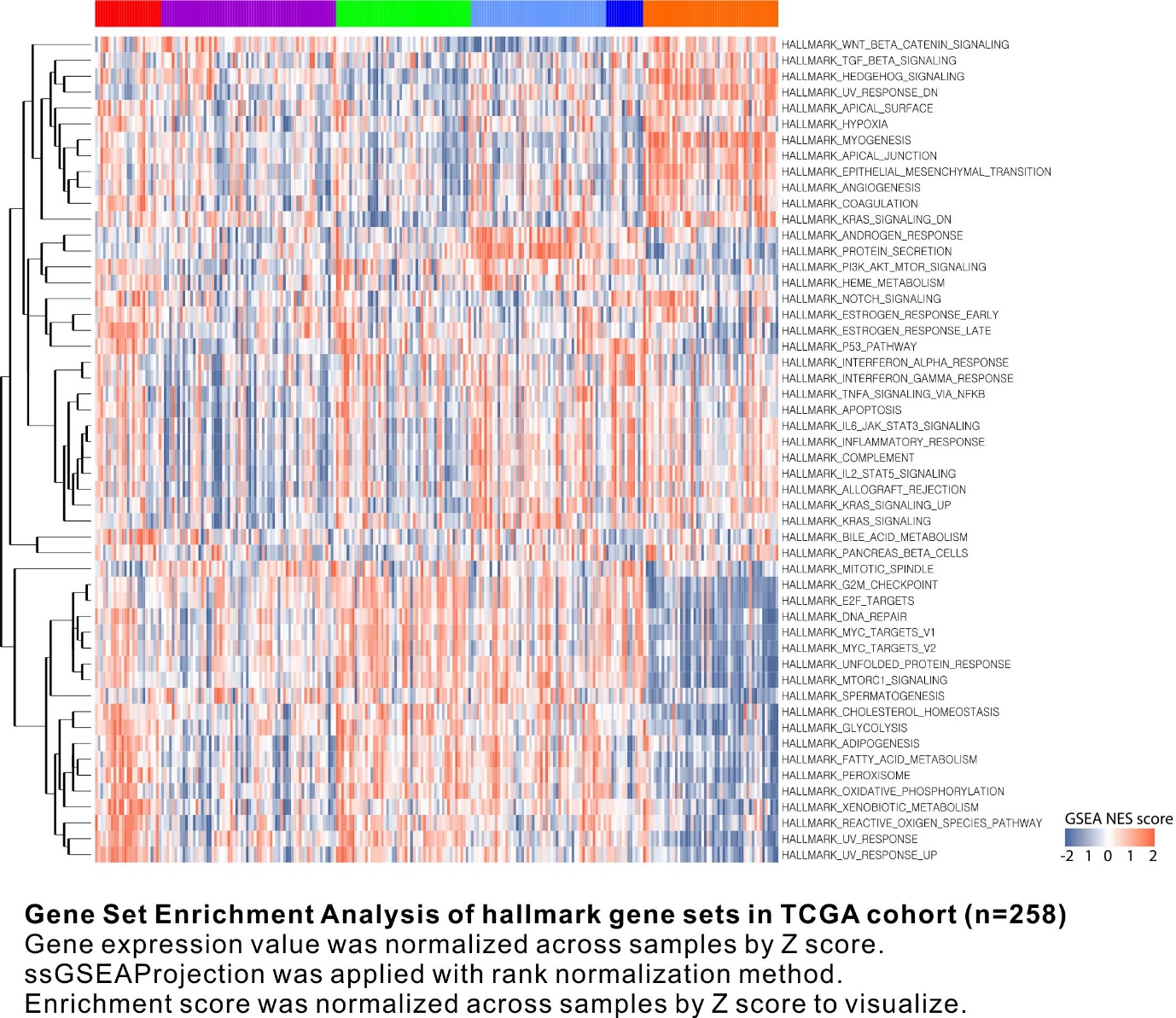
**

**Supplementary Figure S5. Gene set enrichment analysis of hallmark gene sets in the TCGA cohort (*n* = 258)**

The ssGSEA projection was applied using the rank normalization method. The X-axis is arranged as per the LNC6 subtype, and the Y-axis is arranged as per the hierarchical clustering.

**
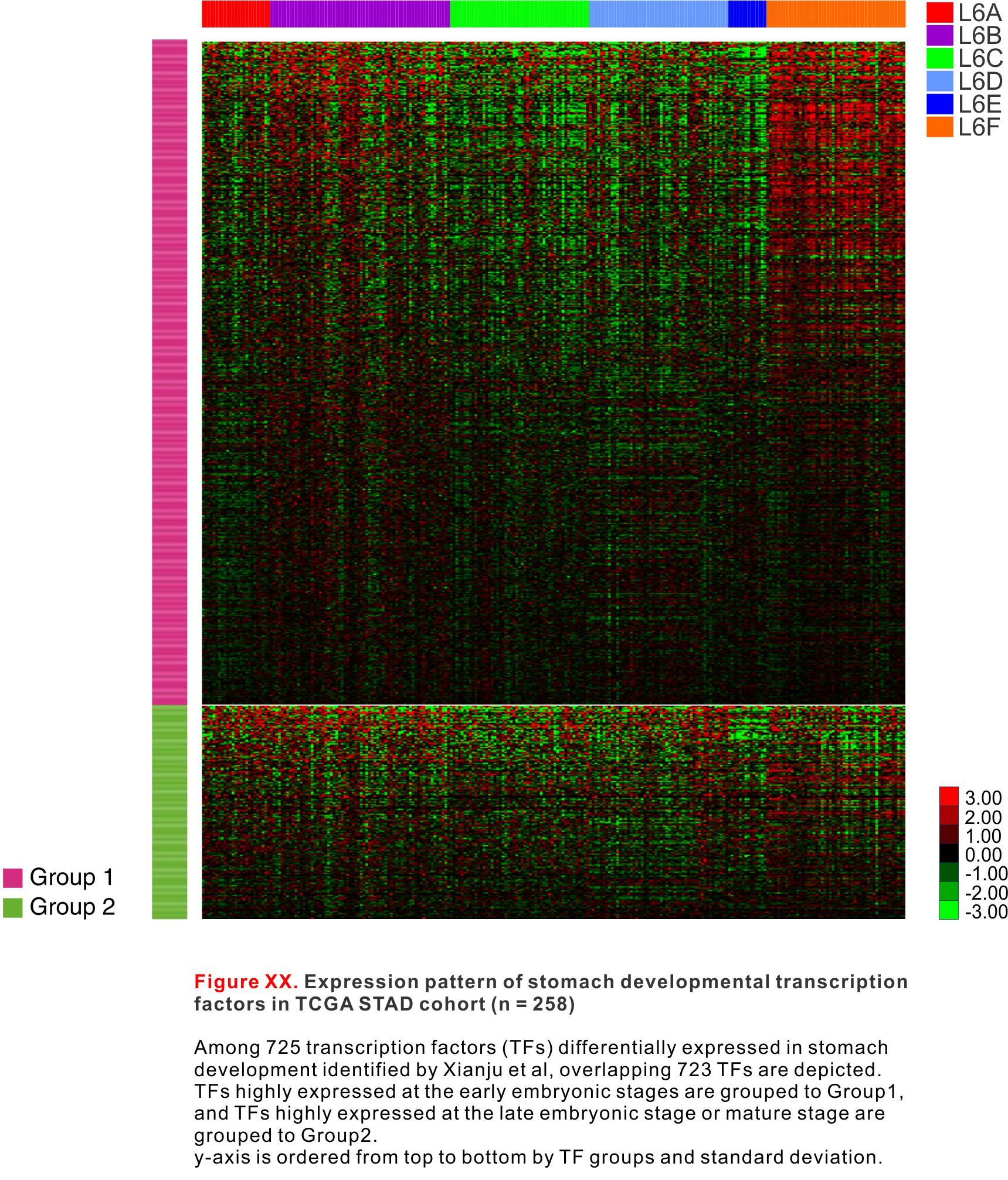
**

**Supplementary Figure S6. Expression pattern of stomach developmental transcription factors in the TCGA cohort (*n* = 258)**

Among 725 stomach developmental transcription factors (TFs) identified at the level of mRNA abundance, 723 TFs, whose expression values are available in the TCGA cohort, were included in the analysis. TFs highly expressed at the early embryonic stages are categorized into group 1, and TFs highly expressed at the late embryonic stage or mature stage are categorized into group 2. The Y-axis is ordered from top to bottom by TF groups and standard deviation across samples.

**
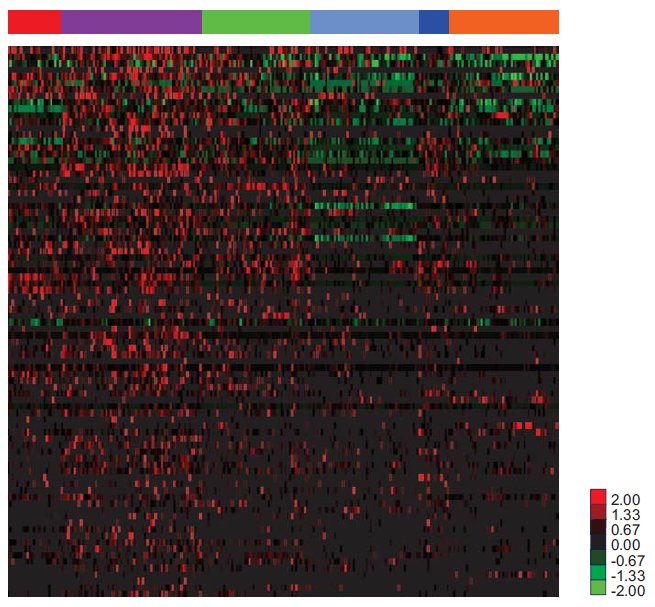
**

**Supplementary Figure S7. Expression pattern of S-phase lncRNAs in the TCGA cohort (*n* = 258)**

Among 1,145 temporally expressed S-phase-enriched lncRNAs, 85 lncRNAs with standard deviation across the TCGA cohort samples greater than 0.3 are depicted.

**Supplementary Table S1. Genomic data used in the validation of clinical association**

| Data set | Platform | Number of patients | Clinical data | Accession number |  |
| --- | --- | --- | --- | --- | --- |
| Korea | Illumina V3 | 267 | Yes | GSE13861 GSE26942 |  |
| Samsung | Illumina V3 | 432 | Yes | GSE26253 |  |
| Shanxi | Affymetrix U133A | 134 | Yes | GSE29272 |  |
| ACRG | Affymetrix U133plus 2 | 300 | Yes | GSE66229 |  |
| KNCC | Affymetrix U133A2 | 167 | Yes | GSE14210 |  |
| Yonsei | Illumina V3 | 433 | Yes | GSE84437 |  |
| Singapore | Affymetrix U133plus 2 | 200 | Yes | GSE15459 |  |
| Total |  | 1,933 |  |  |  |

**Supplementary Table S2. Pairwise comparisons between LNC6 subtypes using log-rank test of overall survival and recurrence-free survival in the test cohorts (*n* = 1,933). P-value adjusted for multiple testing correction using Benjamini-Hochberg method.**

| **Overall survival** | **L6A** | **L6B** | **L6C** | **L6D** | **L6E** |
| --- | --- | --- | --- | --- | --- |
| **L6B** | 0.38183 | - | - | - | - |
| **L6C** | 0.01402 | 0.00038 | - | - | - |
| **L6D** | 0.38183 | 0.86887 | 0.0351 | - | - |
| **L6E** | 0.0351 | 0.05825 | 0.64907 | 0.09218 | - |
| **L6F** | 0.86887 | 0.09017 | 7.40E-08 | 0.29827 | 0.01402 |

| **Recurrence-free survival** | **L6A** | **L6B** | **L6C** | **L6D** | **L6E** |
| --- | --- | --- | --- | --- | --- |
| **L6B** | 0.74 | - | - | - | - |
| **L6C** | 0.323 | 0.051 | - | - | - |
| **L6D** | 0.731 | 0.822 | 0.473 | - | - |
| **L6E** | 0.592 | 0.518 | 0.917 | 0.731 | - |
| **L6F** | 0.74 | 0.039 | 1.00E-05 | 0.235 | 0.232 |

**Supplementary Table S3. Demographic and** **clinical characteristics of the TCGA cohort patients, stratified by the LNC6 subtypes. *P-value* from the Pearson's Chi-squared test.**

| **LNC6** | **L6A** | **L6B** | **L6C** | **L6D** | **L6E** | **L6F** | ***P-value*** |
| --- | --- | --- | --- | --- | --- | --- | --- |
| Age, mean  (SD) | 66.2 (10.3) | 66.2  (9.7) | 68.5 (10.0) | 68.9  (9.9) | 62.6 (12.3) | 60.2 (10.5) |  |
| Male | 21/25 (84%) | 45/66 (68%) | 24/51 (47%) | 26/51 (51%) | 14/14 (100%) | 29/51 (57%) | 0.0004 |
| Western origin | 24/25 (96%) | 47/66 (71%) | 29/51 (57%) | 47/51 (92%) | 9/14 (64%) | 37/51 (73%) | 0.0002 |
| Proximal location | 9/25 (36%) | 14/66 (21%) | 9/51 (18%) | 8/43 (19%) | 3/14 (21%) | 5/51 (10%) | 0.2899 |
| T3/4 | 7/25 (28%) | 48/66 (73%) | 38/50 (76%) | 24/44 (55%) | 13/14 (93%) | 44/51 (86%) | 0.0304 |
| N1-3 | 18/25 (72%) | 42/66 (64%) | 26/50 (52%) | 31/41 (76%) | 10/14 (71%) | 35/50 (70%) | 0.1949 |
| M1 | 0/25 (0%) | 4/62 (6%) | 1/48 (2%) | 7/48 (15%) | 1/12 (8%) | 3/51 (6%) | 0.0873 |
| AJCC Stage III/IV | 8/25 (32%) | 30/66 (45%) | 16/51 (31%) | 21/51 (41%) | 8/14 (57%) | 31/51 (61%) | 0.1133 |
| Histologic Grade 3 | 12/25 (48%) | 34/64 (53%) | 32/51 (63%) | 31/51 (61%) | 14/14 (100%) | 43/48 (90%) | <0.0001 |
| Lauren Diffuse type | 5/25 (20%) | 7/66 (11%) | 3/51 (6%) | 11/51 (23%) | 3/13 (23%) | 34/49 (69%) | <0.0001 |

**Supplementary Table S4. Subtype-specific lncRNAs**

| **ENSEMBL ID** | **Gene name**  **(GENCODE v19)** | **Subtype** | **Ratio (subtype/others)** |
| --- | --- | --- | --- |
| ENSG00000269900.2 | RMRP | L6A | 2.01 |
| ENSG00000242125.2 | SNHG3 | L6A | 1.37 |
| ENSG00000271824.1 | AC009014.3 | L6A | 1.16 |
| ENSG00000126005.11 | MMP24-AS1 | L6A | 0.97 |
| ENSG00000233621.1 | RP11-422J8.1 | L6A | 0.89 |
| ENSG00000229953.1 | RP11-284F21.7 | L6A | 0.88 |
| ENSG00000260704.1 | LINC00543 | L6A | 0.79 |
| ENSG00000175701.6 | LINC00116 | L6A | 0.77 |
| ENSG00000258940.2 | RP11-407N17.5 | L6A | 0.64 |
| ENSG00000226330.1 | RP11-739N20.2 | L6A | 0.61 |
| ENSG00000268516.1 | CTD-3138B18.5 | L6A | -0.26 |
| ENSG00000272645.1 | RP11-504P24.8 | L6A | -0.31 |
| ENSG00000269958.1 | RP11-73M18.8 | L6A | -0.49 |
| ENSG00000267449.1 | RP11-264B14.2 | L6A | -0.54 |
| ENSG00000188206.5 | HNRNPU-AS1 | L6A | -0.58 |
| ENSG00000257621.3 | RP11-349A22.5 | L6A | -0.61 |
| ENSG00000273014.1 | RP11-225B17.2 | L6A | -0.77 |
| ENSG00000229152.1 | ANKRD10-IT1 | L6A | -0.91 |
| ENSG00000259865.1 | RP11-488L18.10 | L6A | -0.96 |
| ENSG00000268205.1 | CTC-444N24.11 | L6A | -0.99 |
| ENSG00000245532.4 | NEAT1 | L6A | -1.14 |
| ENSG00000267207.1 | RP11-264B14.1 | L6A | -1.30 |
| ENSG00000251562.3 | MALAT1 | L6A | -1.39 |
| ENSG00000264940.2 | SNORD3C | L6A | -1.50 |
| ENSG00000130600.11 | H19 | L6B | 2.24 |
| ENSG00000260032.1 | LINC00657 | L6B | 1.58 |
| ENSG00000269972.1 | RP3-430N8.10 | L6B | 1.27 |
| ENSG00000234678.1 | RP11-465N4.4 | L6B | 1.24 |
| ENSG00000269987.1 | RP3-430N8.11 | L6B | 1.19 |
| ENSG00000253352.4 | TUG1 | L6B | 1.15 |
| ENSG00000232803.1 | RP11-93B14.5 | L6B | 1.07 |
| ENSG00000254635.1 | WAC-AS1 | L6B | 1.00 |
| ENSG00000234771.2 | RP11-395P17.3 | L6B | 0.97 |
| ENSG00000270580.1 | RP11-1186N24.5 | L6B | 0.86 |
| ENSG00000234072.1 | AC074117.10 | L6B | 0.84 |
| ENSG00000260766.1 | RP11-226L15.5 | L6B | 0.81 |
| ENSG00000228989.1 | AC133528.2 | L6B | 0.77 |
| ENSG00000206195.6 | AP000525.9 | L6B | 0.74 |
| ENSG00000238035.4 | AC138035.2 | L6B | 0.74 |
| ENSG00000247228.2 | RP11-296I10.3 | L6B | 0.73 |
| ENSG00000267100.1 | ILF3-AS1 | L6B | 0.73 |
| ENSG00000253738.1 | GS1-251I9.4 | L6B | 0.73 |
| ENSG00000226696.1 | LENG8-AS1 | L6B | 0.73 |
| ENSG00000225484.2 | RP11-773D16.1 | L6B | 0.69 |
| ENSG00000188185.7 | LINC00265 | L6B | 0.69 |
| ENSG00000272993.1 | RP11-196G18.24 | L6B | 0.69 |
| ENSG00000231826.1 | AC016735.2 | L6B | 0.66 |
| ENSG00000269990.1 | CTD-3074O7.12 | L6B | 0.66 |
| ENSG00000240731.1 | RP5-890O3.9 | L6B | 0.65 |
| ENSG00000231074.4 | HCG18 | L6B | 0.65 |
| ENSG00000273071.1 | RP11-337C18.10 | L6B | 0.65 |
| ENSG00000177337.3 | DLGAP1-AS1 | L6B | 0.63 |
| ENSG00000261455.1 | LINC01003 | L6B | 0.61 |
| ENSG00000226752.3 | PSMD5-AS1 | L6B | 0.59 |
| ENSG00000259943.1 | RP1-39G22.7 | L6B | 0.58 |
| ENSG00000255503.1 | RP11-113K21.4 | L6B | 0.58 |
| ENSG00000228315.7 | GUSBP11 | L6B | 0.58 |
| ENSG00000240618.1 | RP11-206L10.5 | L6B | 0.57 |
| ENSG00000273000.1 | KB-1572G7.2 | L6B | 0.56 |
| ENSG00000270346.1 | RP1-90J20.12 | L6B | 0.53 |
| ENSG00000225210.5 | AL589743.1 | L6B | 0.52 |
| ENSG00000244306.5 | CTD-2314B22.3 | L6B | 0.51 |
| ENSG00000230724.5 | LINC01001 | L6B | 0.50 |
| ENSG00000261716.1 | RP11-196G18.22 | L6B | 0.43 |
| ENSG00000270157.1 | RP5-894A10.6 | L6B | 0.43 |
| ENSG00000229539.1 | RP11-119B16.2 | L6B | 0.42 |
| ENSG00000232640.1 | RP1-266L20.2 | L6B | 0.42 |
| ENSG00000246067.3 | RAB30-AS1 | L6B | 0.41 |
| ENSG00000273179.1 | RP11-20I20.4 | L6B | 0.41 |
| ENSG00000229043.2 | AC091729.9 | L6B | 0.39 |
| ENSG00000235477.2 | RP11-122G18.5 | L6B | 0.37 |
| ENSG00000225791.2 | TRAM2-AS1 | L6B | 0.34 |
| ENSG00000260233.2 | SSSCA1-AS1 | L6B | 0.32 |
| ENSG00000237094.7 | RP4-669L17.10 | L6B | 0.32 |
| ENSG00000272872.1 | LL22NC03-N14H11.1 | L6B | 0.31 |
| ENSG00000233013.4 | FAM157B | L6B | 0.31 |
| ENSG00000245849.2 | RAD51-AS1 | L6B | 0.31 |
| ENSG00000270015.1 | RP11-540B6.6 | L6B | 0.29 |
| ENSG00000235919.3 | ASH1L-AS1 | L6B | 0.28 |
| ENSG00000227719.1 | AC006042.6 | L6B | 0.27 |
| ENSG00000247400.3 | DNAJC3-AS1 | L6B | 0.27 |
| ENSG00000215394.4 | BMS1P18 | L6B | 0.25 |
| ENSG00000230177.1 | RP5-1112D6.4 | L6B | 0.25 |
| ENSG00000249592.1 | RP11-440L14.1 | L6B | 0.24 |
| ENSG00000179523.4 | EIF3J-AS1 | L6B | 0.23 |
| ENSG00000271975.1 | RP11-383J24.6 | L6B | 0.21 |
| ENSG00000261172.1 | RP11-356C4.5 | L6B | 0.19 |
| ENSG00000247137.4 | RP11-727A23.5 | L6B | 0.18 |
| ENSG00000236859.2 | AC018737.1 | L6B | 0.16 |
| ENSG00000260276.1 | RP11-77H9.2 | L6B | 0.16 |
| ENSG00000206573.4 | SETD5-AS1 | L6B | 0.15 |
| ENSG00000263934.2 | SNORD3A | L6C | 1.59 |
| ENSG00000236081.1 | AC074389.9 | L6C | 1.30 |
| ENSG00000250920.1 | RP11-297P16.4 | L6C | 1.25 |
| ENSG00000260552.1 | RP11-49I11.1 | L6C | 0.82 |
| ENSG00000257084.1 | U47924.27 | L6C | 0.81 |
| ENSG00000240990.5 | HOXA11-AS | L6C | 0.67 |
| ENSG00000256940.1 | RP11-783K16.5 | L6C | 0.63 |
| ENSG00000243766.3 | HOTTIP | L6C | 0.48 |
| ENSG00000245694.4 | CRNDE | L6C | 0.47 |
| ENSG00000204528.3 | PSORS1C3 | L6C | 0.39 |
| ENSG00000228630.1 | HOTAIR | L6C | 0.36 |
| ENSG00000247095.2 | MIR210HG | L6C | 0.26 |
| ENSG00000269867.1 | CTD-2583A14.8 | L6C | 0.11 |
| ENSG00000273142.1 | RP11-458F8.4 | L6C | -0.01 |
| ENSG00000267394.1 | CTB-175E5.7 | L6C | -0.19 |
| ENSG00000267776.1 | AC006116.24 | L6C | -0.31 |
| ENSG00000236933.1 | RP11-439A17.7 | L6C | -0.36 |
| ENSG00000245060.2 | LINC00847 | L6C | -0.44 |
| ENSG00000249456.1 | RP11-298J20.4 | L6C | -0.47 |
| ENSG00000238039.1 | AF011889.2 | L6C | -0.49 |
| ENSG00000232593.2 | RP11-258C19.5 | L6D | 0.45 |
| ENSG00000237250.3 | RP11-193H5.1 | L6D | 0.24 |
| ENSG00000230555.2 | RP11-517P14.2 | L6D | -0.12 |
| ENSG00000214719.7 | AC005562.1 | L6D | -0.12 |
| ENSG00000261801.1 | LOXL1-AS1 | L6D | -0.15 |
| ENSG00000233903.2 | Z83851.4 | L6D | -0.16 |
| ENSG00000230513.1 | THAP7-AS1 | L6D | -0.18 |
| ENSG00000235314.1 | LINC00957 | L6D | -0.18 |
| ENSG00000223813.2 | AC007255.8 | L6D | -0.20 |
| ENSG00000273145.1 | CITF22-92A6.1 | L6D | -0.21 |
| ENSG00000268087.1 | CTC-429P9.2 | L6D | -0.22 |
| ENSG00000267322.1 | SCARNA17 | L6D | -0.26 |
| ENSG00000224914.2 | LINC00863 | L6D | -0.26 |
| ENSG00000267904.1 | CTC-429P9.5 | L6D | -0.27 |
| ENSG00000196810.4 | CTBP1-AS2 | L6D | -0.27 |
| ENSG00000259806.2 | CTD-2196E14.4 | L6D | -0.28 |
| ENSG00000270017.1 | CTD-2576F9.2 | L6D | -0.29 |
| ENSG00000263327.2 | TAPT1-AS1 | L6D | -0.35 |
| ENSG00000245937.3 | CTC-228N24.3 | L6D | -0.36 |
| ENSG00000175772.10 | AC112229.7 | L6D | -0.37 |
| ENSG00000271359.1 | RP11-84C13.1 | L6D | -0.41 |
| ENSG00000269481.1 | CTD-2521M24.6 | L6D | -0.47 |
| ENSG00000273015.1 | LINC00938 | L6D | -0.49 |
| ENSG00000272667.1 | RP11-395A13.2 | L6D | -0.50 |
| ENSG00000272447.1 | RP11-182L21.6 | L6D | -0.53 |
| ENSG00000267080.1 | ASB16-AS1 | L6D | -0.57 |
| ENSG00000253982.1 | CTD-2336O2.1 | L6D | -0.57 |
| ENSG00000239569.2 | KMT2E-AS1 | L6D | -0.59 |
| ENSG00000236618.2 | PITPNA-AS1 | L6D | -0.60 |
| ENSG00000163597.10 | SNHG16 | L6D | -0.60 |
| ENSG00000254911.2 | SCARNA9 | L6D | -0.61 |
| ENSG00000270081.1 | RP5-935K16.1 | L6D | -0.64 |
| ENSG00000226137.3 | BAIAP2-AS1 | L6D | -0.66 |
| ENSG00000233368.2 | RP11-277L2.3 | L6D | -0.68 |
| ENSG00000264575.1 | LINC00526 | L6D | -0.68 |
| ENSG00000265479.1 | DTX2P1-UPK3BP1-PMS2P11 | L6D | -0.69 |
| ENSG00000265091.1 | RP11-835E18.5 | L6D | -0.69 |
| ENSG00000257167.2 | TMPO-AS1 | L6D | -0.72 |
| ENSG00000204054.7 | LINC00963 | L6D | -0.72 |
| ENSG00000236537.1 | RP11-732M18.3 | L6D | -0.73 |
| ENSG00000270103.2 | RNU11 | L6D | -0.73 |
| ENSG00000270170.1 | NCBP2-AS2 | L6D | -0.73 |
| ENSG00000232533.1 | AC093673.5 | L6D | -0.74 |
| ENSG00000262477.1 | AC021224.1 | L6D | -0.75 |
| ENSG00000233223.2 | AC113189.5 | L6D | -0.75 |
| ENSG00000255198.3 | SNHG9 | L6D | -0.77 |
| ENSG00000214783.5 | POLR2J4 | L6D | -0.79 |
| ENSG00000270726.1 | AJ271736.10 | L6D | -0.81 |
| ENSG00000229874.2 | RP11-312O7.2 | L6D | -0.88 |
| ENSG00000273432.1 | RP5-1165K10.2 | L6D | -0.95 |
| ENSG00000261512.2 | RP11-46D6.1 | L6D | -1.01 |
| ENSG00000270066.2 | SCARNA2 | L6D | -1.39 |
| ENSG00000272933.1 | RP11-47A8.5 | L6D | -1.62 |
| ENSG00000258486.2 | RN7SL1 | L6D | -2.31 |
| ENSG00000259001.2 | RPPH1 | L6D | -2.48 |
| ENSG00000250742.1 | RP11-834C11.4 | L6E | 2.93 |
| ENSG00000256039.1 | RP11-291B21.2 | L6E | 1.04 |
| ENSG00000235576.1 | AC092580.4 | L6E | 0.96 |
| ENSG00000253838.1 | RP11-44K6.2 | L6E | 0.90 |
| ENSG00000261520.1 | DLGAP1-AS5 | L6E | 0.82 |
| ENSG00000132832.5 | RP11-445H22.3 | L6E | 0.74 |
| ENSG00000261971.2 | RP11-473M20.7 | L6E | 0.64 |
| ENSG00000227619.1 | RP11-492E3.2 | L6E | 0.57 |
| ENSG00000273290.1 | CTC-297N7.8 | L6E | 0.56 |
| ENSG00000258521.1 | RP11-638I2.9 | L6E | 0.55 |
| ENSG00000239213.1 | RP11-85F14.5 | L6E | 0.52 |
| ENSG00000259834.1 | RP11-284N8.3 | L6E | 0.46 |
| ENSG00000229950.1 | TFAP2A-AS1 | L6E | 0.46 |
| ENSG00000267745.1 | RP11-686D22.8 | L6E | 0.44 |
| ENSG00000262222.1 | RP11-876N24.4 | L6E | 0.32 |
| ENSG00000206028.1 | CTA-373H7.7 | L6E | 0.29 |
| ENSG00000254287.1 | RP11-44K6.4 | L6E | 0.27 |
| ENSG00000225783.2 | MIAT | L6E | 0.26 |
| ENSG00000263013.1 | RP11-876N24.5 | L6E | 0.25 |
| ENSG00000203362.2 | RP3-337H4.8 | L6E | 0.22 |
| ENSG00000267074.1 | RP11-1094M14.5 | L6E | 0.21 |
| ENSG00000249746.1 | RP11-254I22.3 | L6E | 0.16 |
| ENSG00000261270.1 | RP11-325K4.3 | L6E | 0.07 |
| ENSG00000235437.3 | RP11-357C3.3 | L6E | -0.79 |
| ENSG00000272620.1 | AFAP1-AS1 | L6E | -0.86 |
| ENSG00000268913.1 | AC026806.2 | L6E | -0.95 |
| ENSG00000238142.1 | RP11-108M9.4 | L6E | -1.02 |
| ENSG00000261123.1 | RP11-304L19.3 | L6E | -1.29 |
| ENSG00000259933.2 | RP11-304L19.1 | L6E | -1.37 |
| ENSG00000272763.1 | RP11-357H14.17 | L6E | -1.43 |
| ENSG00000272761.1 | RP11-572C15.6 | L6F | 2.02 |
| ENSG00000269936.2 | MIR145 | L6F | 1.72 |
| ENSG00000203706.4 | SERTAD4-AS1 | L6F | 1.17 |
| ENSG00000268388.1 | FENDRR | L6F | 1.07 |
| ENSG00000255248.2 | RP11-166D19.1 | L6F | 1.04 |
| ENSG00000249669.3 | MIR143HG | L6F | 0.92 |
| ENSG00000237125.4 | HAND2-AS1 | L6F | 0.77 |
| ENSG00000224958.1 | PGM5-AS1 | L6F | 0.76 |
| ENSG00000267047.1 | RP11-589P10.7 | L6F | 0.74 |
| ENSG00000166770.6 | ZNF667-AS1 | L6F | 0.66 |
| ENSG00000261625.1 | RP11-554A11.4 | L6F | 0.60 |
| ENSG00000253864.1 | AC131025.8 | L6F | 0.59 |
| ENSG00000250734.2 | RP11-404E16.1 | L6F | 0.58 |
| ENSG00000234638.1 | AC053503.6 | L6F | 0.47 |
| ENSG00000180139.10 | ACTA2-AS1 | L6F | 0.46 |
| ENSG00000235501.1 | RP4-639F20.1 | L6F | 0.44 |
| ENSG00000269186.1 | LINC01082 | L6F | 0.43 |
| ENSG00000230148.4 | HOXB-AS1 | L6F | 0.42 |
| ENSG00000230630.1 | DNM3OS | L6F | 0.39 |
| ENSG00000272755.1 | RP11-326G21.1 | L6F | 0.37 |
| ENSG00000228221.1 | LINC00578 | L6F | 0.37 |
| ENSG00000225986.1 | RP3-340N1.5 | L6F | 0.33 |
| ENSG00000262879.1 | RP11-156P1.3 | L6F | 0.32 |
| ENSG00000259248.1 | USP3-AS1 | L6F | 0.27 |
| ENSG00000234456.3 | MAGI2-AS3 | L6F | 0.27 |
| ENSG00000258441.1 | LINC00641 | L6F | 0.26 |
| ENSG00000261534.1 | RP11-244O19.1 | L6F | 0.26 |
| ENSG00000269044.1 | CTC-429P9.3 | L6F | 0.24 |
| ENSG00000255455.2 | RP11-890B15.3 | L6F | 0.23 |
| ENSG00000267532.2 | MIR497HG | L6F | 0.21 |
| ENSG00000250360.1 | CTD-2089N3.1 | L6F | 0.21 |
| ENSG00000267082.1 | CTC-510F12.2 | L6F | 0.18 |
| ENSG00000224739.2 | AC016735.1 | L6F | -0.18 |
| ENSG00000260711.1 | RP11-747H7.3 | L6F | -0.18 |
| ENSG00000234286.1 | AC006026.13 | L6F | -0.20 |
| ENSG00000238164.2 | RP3-395M20.8 | L6F | -0.21 |
| ENSG00000267751.1 | AC009005.2 | L6F | -0.22 |
| ENSG00000269609.1 | RP11-18I14.10 | L6F | -0.22 |
| ENSG00000260196.1 | RP1-239B22.5 | L6F | -0.22 |
| ENSG00000235823.1 | LINC00263 | L6F | -0.23 |
| ENSG00000253716.1 | RP13-582O9.5 | L6F | -0.26 |
| ENSG00000231770.1 | TMEM44-AS1 | L6F | -0.27 |
| ENSG00000214293.4 | RSBN1L-AS1 | L6F | -0.29 |
| ENSG00000225138.3 | CTD-2228K2.7 | L6F | -0.32 |
| ENSG00000234155.1 | RP11-30P6.6 | L6F | -0.34 |
| ENSG00000251603.1 | RP11-164P12.4 | L6F | -0.34 |
| ENSG00000254837.1 | AP001372.2 | L6F | -0.40 |
| ENSG00000227036.2 | LINC00511 | L6F | -0.43 |
| ENSG00000234608.3 | MAPKAPK5-AS1 | L6F | -0.44 |
| ENSG00000175061.13 | C17orf76-AS1 | L6F | -0.45 |
| ENSG00000177410.8 | ZFAS1 | L6F | -0.51 |
| ENSG00000233834.2 | AC005083.1 | L6F | -0.52 |
| ENSG00000268006.1 | PTOV1-AS1 | L6F | -0.52 |
| ENSG00000247271.2 | ZBED5-AS1 | L6F | -0.55 |
| ENSG00000232677.2 | LINC00665 | L6F | -0.61 |
| ENSG00000196696.8 | PDXDC2P | L6F | -0.64 |
| ENSG00000232445.1 | RP11-132A1.4 | L6F | -0.67 |
| ENSG00000232956.4 | SNHG15 | L6F | -0.71 |
| ENSG00000261183.1 | RP11-532F12.5 | L6F | -0.77 |
| ENSG00000272141.1 | RP11-465B22.8 | L6F | -0.78 |
| ENSG00000196756.7 | SNHG17 | L6F | -0.82 |
| ENSG00000226950.2 | DANCR | L6F | -0.82 |
| ENSG00000261373.1 | VPS9D1-AS1 | L6F | -0.91 |
| ENSG00000224259.1 | RP11-48O20.4 | L6F | -1.05 |
| ENSG00000203499.6 | FAM83H-AS1 | L6F | -1.26 |
| ENSG00000259187.1 | CTD-2008A1.1 | L6F | -1.55 |

**Supplementary Table S5. Ingenuity pathway analysis (IPA) of genes differentially expressed in each LNC6 subtype**

| **LNC6** | **Canonical pathways** | **-log(p-value)** | **zScore** | **Ratio** |
| --- | --- | --- | --- | --- |
|  | Sumoylation Pathway | 2.96 | -1.94 | 15.6% |
|  | DNA Double-Strand Break Repair by Non-Homologous End Joining | 2.89 | NA | 35.7% |
| L6A | Stearate Biosynthesis I (Animals) | 2.80 | 1.67 | 20.5% |
|  | EIF2 Signaling | 2.50 | -2.84 | 11.4% |
|  | Acyl-CoA Hydrolysis | 2.27 | 0.00 | 33.3% |
|  | Type I Diabetes Mellitus Signaling | 4.07 | -1.63 | 8.1% |
|  | Cytotoxic T Lymphocyte-mediated Apoptosis of Target Cells | 3.81 | -2.00 | 15.6% |
| L6B | Antigen Presentation Pathway | 3.45 | NA | 13.2% |
|  | CD28 Signaling in T Helper Cells | 3.45 | -2.00 | 6.7% |
|  | Nur77 Signaling in T Lymphocytes | 3.41 | NA | 10.2% |
|  | Regulation of Actin-based Motility by Rho | 4.32 | 1.00 | 10.1% |
|  | RhoGDI Signaling | 3.88 | 0.30 | 6.9% |
| L6C | Glucocorticoid Receptor Signaling | 2.99 | NA | 4.6% |
|  | Integrin Signaling | 2.96 | -0.30 | 5.4% |
|  | GP6 Signaling Pathway | 2.96 | -2.33 | 6.7% |
|  | Protein Ubiquitination Pathway | 5.43 | NA | 15.5% |
|  | Estrogen Receptor Signaling | 4.84 | NA | 18.7% |
| L6D | Sirtuin Signaling Pathway | 4.62 | -1.33 | 14.4% |
|  | Cleavage and Polyadenylation of Pre-mRNA | 4.00 | NA | 50.0% |
|  | mTOR Signaling | 3.86 | 0.00 | 14.9% |
|  | MSP-RON Signaling Pathway | 4.25 | NA | 17.6% |
|  | Calcium Transport I | 4.24 | 0.45 | 50.0% |
| L6E | Sperm Motility | 3.76 | -1.60 | 13.3% |
|  | Interferon Signaling | 3.54 | 2.12 | 22.2% |
|  | Endothelin-1 Signaling | 3.49 | -1.09 | 11.1% |
|  | Axonal Guidance Signaling | 10.60 | NA | 20.1% |
|  | cAMP-mediated signaling | 10.50 | 5.53 | 25.6% |
| L6F | Hepatic Fibrosis / Hepatic Stellate Cell Activation | 9.48 | NA | 26.3% |
|  | G-Protein Coupled Receptor Signaling | 8.69 | NA | 22.3% |
|  | Gαi Signaling | 7.44 | 2.83 | 27.9% |

| **LNC6** | **Upstream regulator** | **p-value of overlap** | **Predicted Activation State** | **Activation z-score** |
| --- | --- | --- | --- | --- |
|  | HNF4A | 8.92E-08 |  | 1.844 |
|  | CD24 | 1.49E-06 | Inhibited | -4.126 |
| L6A | CST5 | 1.21E-03 |  | 1.768 |
|  | ESR1 | 1.41E-03 | Inhibited | -5.532 |
|  | TCOF1 | 2.00E-03 |  |  |
|  | SAFB | 2.81E-05 |  | 1.673 |
|  | interferon beta-1a | 3.47E-05 |  |  |
| L6B | Sod | 1.45E-04 | Activated | 2 |
|  | EBI3 | 2.28E-04 |  | -0.685 |
|  | CIITA | 4.81E-04 |  | -0.41 |
|  | ERBB2 | 2.91E-05 |  | -1.327 |
|  | Rhox4b (includes others) | 1.36E-04 |  |  |
| L6C | Histone h3 | 2.59E-04 |  |  |
|  | Ctbp | 3.36E-04 |  |  |
|  | MM-401 | 3.70E-04 |  |  |
|  | HNF4A | 3.68E-14 |  | -1.604 |
|  | mir-149 | 4.33E-04 |  |  |
| L6D | miR-16-5p (and other miRNAs w/seed AGCAGCA) | 7.60E-04 | Inhibited | -2.896 |
|  | tunicamycin | 1.27E-03 |  | 1.467 |
|  | ONECUT1 | 1.44E-03 |  |  |
|  | PHF1 | 1.87E-08 | Activated | 2.219 |
|  | KAT6A | 3.16E-08 | Inhibited | -3.231 |
| L6E | CDX2 | 2.13E-07 | Inhibited | -2.762 |
|  | COMMD3-BMI1 | 4.93E-07 | Activated | 2.891 |
|  | STAT5A | 8.44E-07 |  | -1.145 |
|  | TGFB1 | 4.10E-29 | Activated | 7.428 |
|  | ERBB2 | 1.20E-22 | Inhibited | -2.928 |
| L6F | TGFB3 | 1.15E-20 | Activated | 4.795 |
|  | beta-estradiol | 2.69E-19 | Activated | 3.139 |
|  | TWIST1 | 1.17E-18 | Activated | 4.508 |

**Supplementary Table S6. Quantitative real-time RT-PCR primers**

| Gene | Forward Primer | Reverse Primer |
| --- | --- | --- |
| FENDRR | AGAGTGCTTCCACTGCCCTA | CCCATTTGCAAAGGCTACAT |
| MAGI2-AS3 | TGGGTCTGTGCAGAGTTGAG | GCTGGTTATGGCCAATGAGT |
| ACTA2-AS1 | GTGGTTCTGGTTTGCCTGAT | CTGGCCCTGTAACACCAGAT |
| ZNF667-AS1 | GGACACTGTGCAGGATGATG | GGCAAGAATGCTGTGTCTCA |
| RP11-572C15.6 | TCATCCCTCTTCCTTGATGG | ATTGGCAACTTTGGGCTATG |

**Supplementary Table S7. Expression level of lncRNAs relative to GAPDH (log2)**

| **Subtype** | **Cell lines** | **RP11-572C15.6** | **ZNF667-AS1** | **MAGI2-AS3** | **FENDRR** | **ACTA2-AS1** |
| --- | --- | --- | --- | --- | --- | --- |
| EMT | MKN1 | -11.99 | -8.29 | -13.32 | -9.90 | -13.51 |
|  | SNU1750 | -12.87 | -6.46 | -9.94 | -13.52 | -14.29 |
|  | SNU484 | -15.06 | -2.56 | -11.80 | -16.18 | -12.94 |
| non-EMT | YCC3 | -22.26 | -10.62 | -7.66 | -12.98 | -13.92 |
|  | SNU719 | -15.00 | -11.07 | -13.27 | -13.81 | -14.92 |
|  | MKN74 | -16.68 | -11.42 | -16.44 | -15.40 | -14.38 |
|  | Average fold change | 4.67 | 5.27 | 0.77 | 0.86 | 0.83 |
|  | *P*-value (Student’s *t*-test) | 0.12 | 0.04 | 0.79 | 0.68 | 0.17 |

**Material and Methods**

**Genomic and clinical data of the TCGA GC cohort**

Expression profiles for a total of 12,727 lncRNAs from the TCGA stomach adenocarcinoma (STAD) cohort, consisting of 258 tumors, were downloaded from the TANRIC portal, which is based on GENCODE Release 19^2^. mRNA expression profiles of the TCGA STAD cohort were downloaded and processed as described previously^3^. Both expression data were transformed into a log2 base before further analysis. Clinical data of the TCGA STAD cohort were downloaded from the cBioPortal for Cancer Genomics^4^.

**Subtype classification and identification of subtype-specific lncRNAs**

Cluster analysis and visualization of the lncRNA data were performed using Gene Cluster 3.0 and Java Treeview^5^. As a result of hierarchical clustering, the 258 TCGA STAD patients were classified into six clusters, which were named the LNC6 subtypes: L6A (*n* = 25), L6B (*n* = 66), L6C (*n* = 51), L6D (*n* = 51), L6E (*n* = 14), and L6F (*n* = 51). Thereafter, multiple two-class *t*-tests were performed for all possible combinations of the six subtypes to identify subtype-specific lncRNAs. For the selection of subtype L6A, five two-sample *t*-tests (L6A vs. L6B, L6A vs. L6C, L6A vs. L6D, L6A vs. L6E, and L6A vs. L6F comparisons) were conducted. Only lncRNAs with significant differences (*P* < 0.05) in expression in all five possible comparisons were considered subtype-specific.

**Prediction model for LNC6 subtypes**

Subtype-specific mRNA expression signatures were identified using multiple two-class *t*-tests (*P* < 0.001), yielding few hundred genes for each subtype. The top 200 mRNAs were selected for each subtype according to the log ratio. If the number of genes with significant differences in expression was less than 200 in all five possible comparisons, genes with significant differences in four comparisons were considered subtype-specific. To develop a subtype prediction model, a previously developed model using Bayesian compound covariate predictor (BCCP) algorithms was adopted^6,7^. Briefly, gene expression data for 1,200 gene signatures (comprising 200 significant genes for each subtype, as described above) were used to generate the Bayesian probability for each tissue sample belonging to a subtype. Samples in the test cohorts were assigned to one of the six subtypes according to the Bayesian probability scores. BRB-Array Tools (National Institutes of Health) was used for the prediction^8^.

**Genomic and clinical data of test cohorts**

mRNA expression and survival data of seven independent GC cohorts, comprising 1,933 patients, were obtained from the Gene Expression Omnibus (GEO) database of the National Center for Biotechnology Information (NCBI; accession numbers GSE13861, GSE26942, GSE26253, GSE29272, GSE66229, GSE14209, GSE84437, and GSE15459). Datasets description are available in Supplementary Table S1 and previous studies^9-15^. Of the 305 patients with American Joint Commission on Cancer stage II, III, or IV disease without distant metastasis in the merged cohort of GSE13861, GSE15459, and GSE26942, 160 had received standard adjuvant chemotherapy^16,17^.

**LncRNA expression analysis of the immunotherapy cohort and GC cell lines**

LncRNA expression was analyzed from the raw RNA sequencing data from two previous studies^1,18^. In total, 45 specimens from patients with metastatic GC who participated in a phase 2 trial of pembrolizumab and 29 DNA-fingerprinted GC cell lines were used. Reads were aligned to the reference human genome GRCh38 using STAR 2.6.0c^19^, as per the methods used by the International Cancer Genome Consortium. Uniquely mapped reads for each non-coding RNA were calculated using the Rsubread package (ver. 1.34.0)^20^ with GENCODE annotations (Release 22). Fragments per kilobase of transcript per million mapped read values were calculated using R (<https://www.r-project.org/>) according to its definition.

**Pharmacogenomic analysis in GC cell lines**

Cancer Cell Line Encyclopedia (CCLE) RNAseq gene expression data (log2(TPM+1)) was downloaded from DepMap (Public 19Q1)^21^. Area under the dose-response curve (AUC) values were acquired from Genomics of Drug Sensitivity in Cancer Project (GDSC)^22^ for CCLE cell lines tested on 266 drugs and a previous study^23^ for 29 GC cell lines tested on 75 drugs. To identify drugs that could specifically target L6F subtype gastric cancer, spearman correlation tests were assessed between the probability of gastric cancer cell lines belonging to L6F subtype and the AUC values.

**Statistical analysis**

The association of each subtype with overall survival (OS) and recurrence-free survival (RFS) was estimated using Kaplan–Meier plots and log-rank tests. All statistical analyses were conducted in the R language environment (<http://www.r-project.org>).

**Bioinformatics analysis**

Single-sample GSEA (ssGSEA), an extension of Gene Set Enrichment Analysis (GSEA), was performed using GenePattern in the TCGA cohort and the immunotherapy cohort^24-26^. Briefly, this allows calculation of separate enrichment scores for each pairing of a sample and gene set, representing the degree to which the genes in a gene set are coordinately up- or downregulated within a sample. For the expression values, Z-normalization was performed per gene across samples; these values were then ranked by Z scores per sample. A previously defined “hallmark” gene set was used for the analysis of the TCGA cohort^27^. The resulting enrichment scores were normalized across samples by Z score (NES, normalized enrichment score).

Ingenuity pathway analysis (IPA) was used to identify canonical pathways and upstream regulators of each subtype. As IPA requires an adequate number of genes, mRNAs with significant differences (*P* < 0.05) in expression in all five possible comparisons were used for the analysis. Only mRNAs with an absolute log ratio greater than 1 were used for the L6F analysis. The resulting number of genes were 1,576 for L6A, 404 for L6B, 427 for L6C, 1,756 for L6D, and 2,391 for L6F. The analysis was based on the expression log ratio of genes between samples in a subtype and the rest of the samples in the TCGA cohort.

**Other biological characteristics analyses**

The stemness of each subtype was assessed from the expression level of transcription factors (TFs) differentially expressed in mouse stomach developmental stages. Among 725 stomach developmental TFs identified at the mRNA abundance level, 723 TFs, whose expression values are available in the TCGA cohort, were included in the analysis^28^. The cell cycle phase in each subtype was assessed from the expression level of S-phase-enriched lncRNAs, identified by nascent RNA-capture sequencing^29^. Among 1,145 temporally expressed S-phase-enriched lncRNAs, lncRNAs with standard deviation across TCGA cohort samples greater than 0.3 were included in the analysis.

**Cell culture, transfection, and quantitative real-time RT-PCR**

GC cell lines were cultured in RPMI-1640 with 10% fetal bovine serum and penicillin/streptomycin (100 µg/L each) in a humidified incubator containing 5% CO_2_ at 37 °C. ZNF667-AS1 knockdown by siRNA (ThermoFisher Scientific) was performed using the Mirus transfection reagent (Mirus Bio, Madison, WI, USA). Total RNA was extracted from cancer cells using the TRI reagent according to the manufacturer’s instructions (Molecular Research Center Inc., Cincinnati, OH, USA). cDNA was synthesized using M-MLV Reverse Transcriptase (Enzynomics, South Korea). For overexpression of ZNF667-AS1, the sequence of ZNF667-AS1 was synthesized and subcloned into pCMV-AC-GFP vector. BamH1/ Xho1 restriction sites were added at both ends. The recombinant plasmids were extracted and transfected into cancer cells. Gene expression was measured by real-time qPCR on the Eco Real-time PCR system (Illumina, San Diego, CA, USA) using the SYBR Green PCR master mix (ThermoFisher Scientific) and primers are listed in Supplementary Table S2. The relative gene expression was normalized to GAPDH.

**Cell migration, invasion, sphere formation and viability assay**

A 24-well plate with 8-µm pore size chamber inserts (Corning Costar) was used to evaluate the migration and invasion of cancer cells transfected with si-non-target or si-ZNF667-AS1. The number of cells that had migrated or invaded was counted using the EVOS M7000 imaging system (ThermoFisher Scientific). 5 × 10^4^ cells/mL were seeded in 6-well ultra-low adhesion plates (Corning) in DMEM/F12 (GIBCO) with 5% FBS medium supplemented with 10ng/mL EGF< 10ug/mL insulin and 1ug/mL hydrocortisone (GIBCO). 1mL of medium per well were added every 2 days. After 14 days, the circumference of sphere was analyzed by Image J software. Cancer cells transfected with si-non-target or si-ZNF667-AS1 were seeded in 96-well plates (2 × 10^3^ cells/well) overnight before drug treatment, further they were maintained in the presence of drugs for 72 h, before 20 µL CellTiter 96 AQ_ueous_ One Solution (MTS, Promega) was added per well. Plates were incubated for 3 h before absorbance was measured at 490 nm using an ELISA reader (BioTek).

**Immunoblotting**

Cells were lysed in RIPA buffer supplemented with protease inhibitor cocktail and phosphatase inhibitors (Roche). The total protein concentration was determined using the BCA protein assay kit (ThermoFisher Scientific). In total, 10 µg of protein was separated by SDS-PAGE, transferred onto a PVDF membrane (Millipore), and incubated overnight at 4 °C with primary antibodies. Primary antibodies used were: ZNF667 (1:2,000, Abcam, ab106432), N-cadherin (1:1,000, Cell Signaling, 4061S), E-cadherin (1:1,000, Cell Signaling, 14472S), vimentin (1:1,000, Cell Signaling, 5741), and GAPDH (1:1,000, Sigma, G9545). Following five washes with TBS-T, the blot was incubated with horseradish peroxidase-conjugated secondary antibody and visualized by enhanced chemiluminescence detection (ECL plus kit, Pierce).

**Supplementary Discussion**

**Previously reported clinical association of L6C and L6F-specifc lncRNAs**

Five lncRNAs – *HOXA11-AS* ^30^, *HOTTIP* ^31^, *CRNDE* ^32^, *HOTAIR* ^33^, and *MIR210HG* ^34^ – that are specifically upregulated in L6C subtype have been previously associated with chemoresistance in cancers, including resistance to cisplatin and 5-FU in GC. Seven L6F-specific lncRNAs – MIR100HG ^35-37^, HAND2-AS1 ^38,39^, PGM5-AS1 ^40-42^, ACTA2-AS1 ^43^, HOXB-AS1 ^44^, DNM3OS ^45^, MAGI2-AS3 ^46^ – have shown implications in poor clinical outcome, stem-like phenotypes, EMT process, and TGF-β/SMAD signaling. We anticipate further clinical validation and functional evaluation of these lncRNAs.

**Gene set enrichment analysis and ingenuity pathway analysis**

The L6A subtype was characterized by the activation of metabolic pathways, including glycolysis, oxidative phosphorylation, and fatty acid metabolism. Hepatocyte nuclear factor-4α (HNF4α) was predicted to be the most significant upstream regulator of the L6A subtype, consistent with a previous report implicating HNF4α as a metabolic switch in GC^47^. The L6C subtype was characterized by the activation of G2M checkpoint, E2F targets, DNA repair, MYC targets, and MTORC1 signaling. The L6D subtype was characterized by the activation of protein secretion and KRAS signaling; the L6E subtype was characterized by the activation of the interferon response, which supports the active immunity of the L6E subtype. The L6F subtype showed the activation of Wnt/β-catenin and TGF-β signaling, epithelial-mesenchymal transition (EMT), and angiogenesis. Additionally, TGF-β and Twist1, which are the major regulators of EMT, were predicted as upstream regulators of the L6F subtype.

**Drug repurposing approach for the L6F subtype**

There were three drugs that commonly showed specific efficacy to the L6F subtype in pharmacogenomic analyses: YM155, PI-103, and Obatoclax Mesylate. Among them, PI-103 and Obatoclax had previously been tested in another high-throughput drug screen of GC cell lines, and both were effective against the mesenchymal subtype^16^. In addition, YM155 reduced gastric cancer stem cells (CSCs) *in vivo* ^48^, which resonates with the stem-like characteristics of the L6F subtype. Interestingly, PI-103 showed selective efficacy for liver cancer cells that are dependent on oxidative phosphorylation (OXPHOS)^49^, which is in consonance with the dependence of OXPHOS in CSCs ^50^. In line with this, Obatoclax Mesylate selectively eliminated leukemia stem cells by reducing OXPHOS ^51^. These imply that metabolic characteristics of CSCs are represented in lncRNA gene expression, and we expect further studies on lncRNA-mediated metabolic adaptation of CSCs.

1. Lee J, Kim H, Lee JE, et al. Selective Cytotoxicity of the NAMPT Inhibitor FK866 Toward Gastric Cancer Cells With Markers of the Epithelial-Mesenchymal Transition, Due to Loss of NAPRT. *Gastroenterology.* 2018;155(3):799-814 e713.

2. Li J, Han L, Roebuck P, et al. TANRIC: An Interactive Open Platform to Explore the Function of lncRNAs in Cancer. *Cancer Res.* 2015;75(18):3728-3737.

3. Comprehensive molecular characterization of gastric adenocarcinoma. *Nature.* 2014;513(7517):202-209.

4. Cerami E, Gao J, Dogrusoz U, et al. The cBio Cancer Genomics Portal: An Open Platform for Exploring Multidimensional Cancer Genomics Data. 2012;2(5):401-404.

5. Eisen MB, Spellman PT, Brown PO, Botstein D. Cluster analysis and display of genome-wide expression patterns. *Proc Natl Acad Sci U S A.* 1998;95(25):14863-14868.

6. Lee JS, Chu IS, Heo J, et al. Classification and prediction of survival in hepatocellular carcinoma by gene expression profiling. *Hepatology.* 2004;40(3):667-676.

7. Lee JS, Heo J, Libbrecht L, et al. A novel prognostic subtype of human hepatocellular carcinoma derived from hepatic progenitor cells. *Nat Med.* 2006;12(4):410-416.

8. Simon R, Lam A, Li MC, Ngan M, Menenzes S, Zhao Y. Analysis of gene expression data using BRB-ArrayTools. *Cancer Inform.* 2007;3:11-17.

9. Cheong JH, Yang HK, Kim H, et al. Predictive test for chemotherapy response in resectable gastric cancer: a multi-cohort, retrospective analysis. *Lancet Oncol.* 2018;19(5):629-638.

10. Cristescu R, Lee J, Nebozhyn M, et al. Molecular analysis of gastric cancer identifies subtypes associated with distinct clinical outcomes. *Nat Med.* 2015;21(5):449-456.

11. Lee J, Sohn I, Do IG, et al. Nanostring-based multigene assay to predict recurrence for gastric cancer patients after surgery. *PLoS One.* 2014;9(3):e90133.

12. Ooi CH, Ivanova T, Wu J, et al. Oncogenic pathway combinations predict clinical prognosis in gastric cancer. *PLoS Genet.* 2009;5(10):e1000676.

13. Sohn BH, Hwang JE, Jang HJ, et al. Clinical Significance of Four Molecular Subtypes of Gastric Cancer Identified by The Cancer Genome Atlas Project. *Clin Cancer Res.* 2017.

14. Wang G, Hu N, Yang HH, et al. Comparison of global gene expression of gastric cardia and noncardia cancers from a high-risk population in china. *PLoS One.* 2013;8(5):e63826.

15. Kim HK, Choi IJ, Kim CG, et al. A gene expression signature of acquired chemoresistance to cisplatin and fluorouracil combination chemotherapy in gastric cancer patients. *PLoS One.* 2011;6(2):e16694.

16. Lei Z, Tan IB, Das K, et al. Identification of molecular subtypes of gastric cancer with different responses to PI3-kinase inhibitors and 5-fluorouracil. *Gastroenterology.* 2013;145(3):554-565.

17. Oh SC, Sohn BH, Cheong JH, et al. Clinical and genomic landscape of gastric cancer with a mesenchymal phenotype. *Nat Commun.* 2018;9(1):1777.

18. Kim ST, Cristescu R, Bass AJ, et al. Comprehensive molecular characterization of clinical responses to PD-1 inhibition in metastatic gastric cancer. *Nature Medicine.* 2018;24(9):1449-1458.

19. Dobin A, Davis CA, Schlesinger F, et al. STAR: ultrafast universal RNA-seq aligner. *Bioinformatics.* 2013;29(1):15-21.

20. Liao Y, Smyth GK, Shi W. The R package Rsubread is easier, faster, cheaper and better for alignment and quantification of RNA sequencing reads. *Nucleic Acids Res.* 2019.

21. The Cancer Cell Line Encyclopedia C, Stransky N, Ghandi M, et al. Pharmacogenomic agreement between two cancer cell line data sets. *Nature.* 2015;528:84.

22. Iorio F, Knijnenburg TA, Vis DJ, et al. A Landscape of Pharmacogenomic Interactions in Cancer. *Cell.* 2016;166(3):740-754.

23. Lee Y, Lee CE, Oh S, et al. Pharmacogenomic Analysis Reveals CCNA2 as a Predictive Biomarker of Sensitivity to Polo-Like Kinase I Inhibitor in Gastric Cancer. 2020;12(6):1418.

24. Barbie DA, Tamayo P, Boehm JS, et al. Systematic RNA interference reveals that oncogenic KRAS-driven cancers require TBK1. *Nature.* 2009;462(7269):108-112.

25. Reich M, Liefeld T, Gould J, Lerner J, Tamayo P, Mesirov JP. GenePattern 2.0. *Nat Genet.* 2006;38(5):500-501.

26. Subramanian A, Tamayo P, Mootha VK, et al. Gene set enrichment analysis: a knowledge-based approach for interpreting genome-wide expression profiles. *Proc Natl Acad Sci U S A.* 2005;102(43):15545-15550.

27. Liberzon A, Birger C, Thorvaldsdottir H, Ghandi M, Mesirov JP, Tamayo P. The Molecular Signatures Database (MSigDB) hallmark gene set collection. *Cell Syst.* 2015;1(6):417-425.

28. Li X, Zhang C, Gong T, et al. A time-resolved multi-omic atlas of the developing mouse stomach. *Nature Communications.* 2018;9(1):4910.

29. Ali MM, Akhade VS, Kosalai ST, et al. PAN-cancer analysis of S-phase enriched lncRNAs identifies oncogenic drivers and biomarkers. *Nature Communications.* 2018;9(1):883.

30. Zhao X, Li X, Zhou L, et al. LncRNA HOXA11-AS drives cisplatin resistance of human LUAD cells via modulating miR-454-3p/Stat3. *Cancer Sci.* 2018;109(10):3068-3079.

31. Wang SS, Wuputra K, Liu CJ, et al. Oncogenic function of the homeobox A13-long noncoding RNA HOTTIP-insulin growth factor-binding protein 3 axis in human gastric cancer. *Oncotarget.* 2016;7(24):36049-36064.

32. Han P, Li JW, Zhang BM, et al. The lncRNA CRNDE promotes colorectal cancer cell proliferation and chemoresistance via miR-181a-5p-mediated regulation of Wnt/β-catenin signaling. *Mol Cancer.* 2017;16(1):9.

33. Yan J, Dang Y, Liu S, Zhang Y, Zhang G. LncRNA HOTAIR promotes cisplatin resistance in gastric cancer by targeting miR-126 to activate the PI3K/AKT/MRP1 genes. *Tumour Biol.* 2016.

34. Li D, Qian X, Xu P, et al. Identification of lncRNAs and Their Functional Network Associated with Chemoresistance in SW1990/GZ Pancreatic Cancer Cells by RNA Sequencing. *DNA Cell Biol.* 2018;37(10):839-849.

35. Chen D, Sun Y, Yuan Y, et al. miR-100 induces epithelial-mesenchymal transition but suppresses tumorigenesis, migration and invasion. *PLoS Genet.* 2014;10(2):e1004177.

36. Li J, Xu Q, Wang W, Sun S. MIR100HG: a credible prognostic biomarker and an oncogenic lncRNA in gastric cancer. *Biosci Rep.* 2019;39(4).

37. Ottaviani S, Stebbing J, Frampton AE, et al. TGF-β induces miR-100 and miR-125b but blocks let-7a through LIN28B controlling PDAC progression. *Nat Commun.* 2018;9(1):1845.

38. Wang Y, Zhu P, Luo J, et al. LncRNA HAND2-AS1 promotes liver cancer stem cell self-renewal via BMP signaling. *Embo j.* 2019;38(17):e101110.

39. Yang Y, Chen L, Gu J, et al. Recurrently deregulated lncRNAs in hepatocellular carcinoma. *Nat Commun.* 2017;8:14421.

40. Liu W, Liu P, Gao H, Wang X, Yan M. Long non-coding RNA PGM5-AS1 promotes epithelial-mesenchymal transition, invasion and metastasis of osteosarcoma cells by impairing miR-140-5p-mediated FBN1 inhibition. *Mol Oncol.* 2020;14(10):2660-2677.

41. Shen Y, Qi L, Li Y, et al. The Downregulation of lncRNA PGM5-AS1 Inhibits the Proliferation and Metastasis Via Increasing miR-484 Expression in Colorectal Cancer. *Cancer Biother Radiopharm.* 2020.

42. Zhu H, Yu J, Zhu H, Guo Y, Feng S. Identification of key lncRNAs in colorectal cancer progression based on associated protein-protein interaction analysis. *World J Surg Oncol.* 2017;15(1):153.

43. Luo L, Wang M, Li X, et al. A novel mechanism by which ACTA2-AS1 promotes cervical cancer progression: acting as a ceRNA of miR-143-3p to regulate SMAD3 expression. *Cancer Cell Int.* 2020;20:372.

44. Chen X, Li LQ, Qiu X, Wu H. Long non-coding RNA HOXB-AS1 promotes proliferation, migration and invasion of glioblastoma cells via HOXB-AS1/miR-885-3p/HOXB2 axis. *Neoplasma.* 2019;66(3):386-396.

45. Wang S, Ni B, Zhang Z, et al. Long non-coding RNA DNM3OS promotes tumor progression and EMT in gastric cancer by associating with Snail. *Biochem Biophys Res Commun.* 2019;511(1):57-62.

46. Li D, Wang J, Zhang M, et al. LncRNA MAGI2-AS3 Is Regulated by BRD4 and Promotes Gastric Cancer Progression via Maintaining ZEB1 Overexpression by Sponging miR-141/200a. *Mol Ther Nucleic Acids.* 2020;19:109-123.

47. Chang HR, Nam S, Kook MC, et al. HNF4alpha is a therapeutic target that links AMPK to WNT signalling in early-stage gastric cancer. *Gut.* 2016;65(1):19-32.

48. Cheng XJ, Lin JC, Ding YF, Zhu L, Ye J, Tu SP. Survivin inhibitor YM155 suppresses gastric cancer xenograft growth in mice without affecting normal tissues. *Oncotarget.* 2016;7(6):7096-7109.

49. Tan JL, Li F, Yeo JZ, et al. New High-Throughput Screening Identifies Compounds That Reduce Viability Specifically in Liver Cancer Cells That Express High Levels of SALL4 by Inhibiting Oxidative Phosphorylation. *Gastroenterology.* 2019;157(6):1615-1629.e1617.

50. Shin M-K, Cheong J-H. Mitochondria-centric bioenergetic characteristics in cancer stem-like cells. *Archives of Pharmacal Research.* 2019;42(2):113-127.

51. Lagadinou ED, Sach A, Callahan K, et al. BCL-2 inhibition targets oxidative phosphorylation and selectively eradicates quiescent human leukemia stem cells. *Cell Stem Cell.* 2013;12(3):329-341.
